# Seasonal adaptations of the hypothalamo-neurohypophyseal system of the dromedary camel

**DOI:** 10.1371/journal.pone.0216679

**Published:** 2019-06-18

**Authors:** Fatma Zohra Djazouli Alim, Elena V. Romanova, Yea-Ling Tay, Ahmad Yamin bin Abdul Rahman, Kok-Gan Chan, Kar-Wai Hong, Mark Rogers, Bruce R. Southey, Michael P. Greenwood, Andre Souza Mecawi, Mohammad Rais Mustafa, Nicole Mahy, Colin Campbell, José Antunes-Rodrigues, Jonathan V. Sweedler, David Murphy, Charles C. T. Hindmarch

**Affiliations:** 1 Department of Biotechnologies, Faculty of Nature and Life Sciences, Route de soumaa Blida, Algeria; 2 Laboratory of Animal Eco-Biology (LEBA), Normale Superior School of Kouba, Bachir El Ibrahimi, Algeria; 3 Department of Chemistry and the Beckman Institute, University of Illinois Urbana-Champaign, Urbana, Illinois, United States of America; 4 BioEasy Sdn Bhd, Setia Alam, Shah Alam, Selangor Darul Ehsan, Malaysia; 5 International Genome Centre, Jiangsu University, Zhenjiang, China; 6 Division of Genetics and Molecular Biology, Institute of Biological Sciences, Faculty of Science, University of Malaya, Kuala Lumpur, Malaysia; 7 Department of Engineering Mathematics, University of Bristol, Merchant Venturer’s Buliding, Bristol, England; 8 Department of Animal Sciences, University of Illinois Urbana-Champaign, Animal Sciences Laboratory, Urbana, Illinois, United States of America; 9 School of Clinical Sciences, Dorothy Hodgkin Building, University of Bristol, Bristol, England; 10 Department of Physiological Sciences, Institute of Biological and Health Sciences, Federal Rural University of Rio de Janeiro, Seropédica, RJ, Brazil; 11 Department of Physiology, Faculty of Medicine, University of Malaya, Kuala Lumpur, Malaysia; 12 Department of Physiology, Faculty of Medicine of Ribeirão Preto, University of São Paulo, Ribeirão Preto. SP, Brazil; 13 Department of Pharmacology, Faculty of Medicine, University of Malaya, Kuala Lumpur, Malaysia; 14 Departament de Biomedicina, Institut d'Investigacions Biomèdiques August Pi i Sunyer (IDIBAPS), Institut de Neurociències, Universitat de Barcelona and Centro de Investigación Biomédica en Red sobre Enfermedades Neurodegenerativas (CIBERNED), Barcelona, Spain; 15 Queen’s Cardiopulmonary Unit (QCPU), Translational Institute of Medicine (TIME), Department of Medicine, Queen’s University, Kingston, Ontario, Canada; UT Southwestern Medical Center, UNITED STATES

## Abstract

The “ship” of the Arabian and North African deserts, the one-humped dromedary camel (*Camelus dromedarius)* has a remarkable capacity to survive in conditions of extreme heat without needing to drink water. One of the ways that this is achieved is through the actions of the antidiuretic hormone arginine vasopressin (AVP), which is made in a specialised part of the brain called the hypothalamo-neurohypophyseal system (HNS), but exerts its effects at the level of the kidney to provoke water conservation. Interestingly, our electron microscopy studies have shown that the ultrastructure of the dromedary HNS changes according to season, suggesting that in the arid conditions of summer the HNS is in an activated state, in preparation for the likely prospect of water deprivation. Based on our dromedary genome sequence, we have carried out an RNAseq analysis of the dromedary HNS in summer and winter. Amongst the 171 transcripts found to be significantly differentially regulated (>2 fold change, *p* value <0.05) there is a significant over-representation of neuropeptide encoding genes, including that encoding AVP, the expression of which appeared to increase in summer. Identification of neuropeptides in the HNS and analysis of neuropeptide profiles in extracts from individual camels using mass spectrometry indicates that overall AVP peptide levels decreased in the HNS during summer compared to winter, perhaps due to increased release during periods of dehydration in the dry season.

## Introduction

Water balance is aggressively defended in all mammals [1], but this is all the more so in the homeostatic masterpiece that is the dromedary camel, which has a remarkable capacity to thrive in the hot, arid conditions of the Arabian and North African deserts [2–6], and to survive extended periods of dehydration during the summer months [7,8]. The dromedary is thus an ideal model for understanding the genomic and physiological mechanisms that enable mammals to survive in arid regions, and to integrate and reconcile the competing demands of thermoregulation and osmoregulation [9].

Water loss is extremely well tolerated in the dromedary camel; whilst 12% would be fatal to non-desert mammals due to cardiac failure resulting from circulatory disturbance [10], the dromedary can survive up to 30% water loss [11]. However, it is water economy that is vital for survival in the desert, and, in the dromedary camel, this is achieved by minimal evaporative cooling (camels rarely sweat), low urinary output, water extraction from undigested food residues, and variation in body temperature from 34°C at night up to 42°C during the day. This 8°C variation in body temperature allows a 750 kg camel to store 3.9 kJ of heat energy per kg of body weight for each 1C increase in body temperature, which is dissipated at night [12,13]. This mechanism prevents insensible water loss through the secretion of sweat, and corresponds to a saving up to 5 L of water every day.

At the level of the kidney, the dromedary camel produces a low volume of highly concentrated urine, especially following dehydration, as a consequence of the highly efficient reabsorption of water [14]. This is mediated by the actions of the antidiuretic hormone arginine vasopressin (AVP), which is made in a specialised part of the brain called the hypothalamo-neurohypophyseal system (HNS). The HNS consists of the large peptidergic magnocellular neurones of the hypothalamic supraoptic and paraventricular nuclei [15]. The axons of these neurones course though the internal zone of the median eminence to terminations on blood capillaries of the posterior pituitary gland [15]. AVP, and the related hormone oxytocin (OT), are transported down this conduit to storage in posterior pituitary axon terminals until mobilised for secretion into the systemic circulation. Upon release, AVP travels through the blood stream to specific receptor targets located in the kidney where it promotes water reabsorption in the collecting duct [16]. In addition to its well-known roles in parturition and lactation, OT is thought to have natriuretic activity at the level of the kidney [17]. Circulating levels of AVP increase following dehydration in the dromedary camel [14,18], and the sensitivity of the renal response to AVP has been reported to be 100 fold greater in the dromedary camel compared with cattle [19].

We have previously used electron microscopy to characterize the organization of the dromedary neurointermediate lobe of the pituitary, which is the combination of the posterior and intermediate lobes, and we have described how these change with season [20,21]. These studies suggest that in the arid conditions of summer the dromedary’s neurointermediate lobe is in an activated state, in preparation for the likely prospect of water deprivation.

In this study, we have sought to further explore the seasonal plasticity of the dromedary HNS. We compared hydrated dromedary camels from winter and summer seasons in the Algerian Sahara. We used electron microscopy to describe how the ultrastructure of the supraoptic nucleus differs in winter and summer and, in order to understand the genomic basis of HNS seasonal plasticity, we have used RNAseq to catalogue global changes in gene expression in the camel supraoptic nucleus between winter and summer. Finally, we used mass spectrometry to identify neuropeptides and their seasonal differences in the supraoptic nucleus and the neurointermediate pituitary lobe. These analyses required a camel genome sequence.

## Results

### Structural organisation of the dromedary supraoptic nucleus

The dromedary HNS begins at the base of the brain and projects to the posterior lobe of hypophysis (S1 Fig). The brain regions dissected for analysis in this study are illustrated (S1 Fig). At the level of optic chiasm, coronal hypothalamic slices at the rostral median and caudal zones (S2A–S2D Fig) reveal the packed magnocellular neurone cell bodies of the supraoptic nucleus (viewed in Nissl-stained sections; S2A'–S2D' Fig). Magnocelluar neurone cell bodies, glial cells and blood vessels are the most important elements found in the supraoptic nucleus (S3A and S3B Fig). Visualisation of semi-thin sections reveals distinct somatic and dendritic zones of supraoptic nucleus (S3C Fig). The somatic zone is occupied by magnocellular neurons of light and dark appearance (S3C and S3D Fig). Capillaries and glial cells are scattered through the nucleus and are observed close to magnocellular neurones (S3D Fig). Both vasopressinergic (S4A Fig) and oxytocinergic (S4B Fig) magocellular neurones were identified in the dromedary supraoptic nucleus, intermingled throughout the nucleus. Glial processes make up a network between magnocellular neurones and capillaries (S4C Fig).

**Fig 1:**
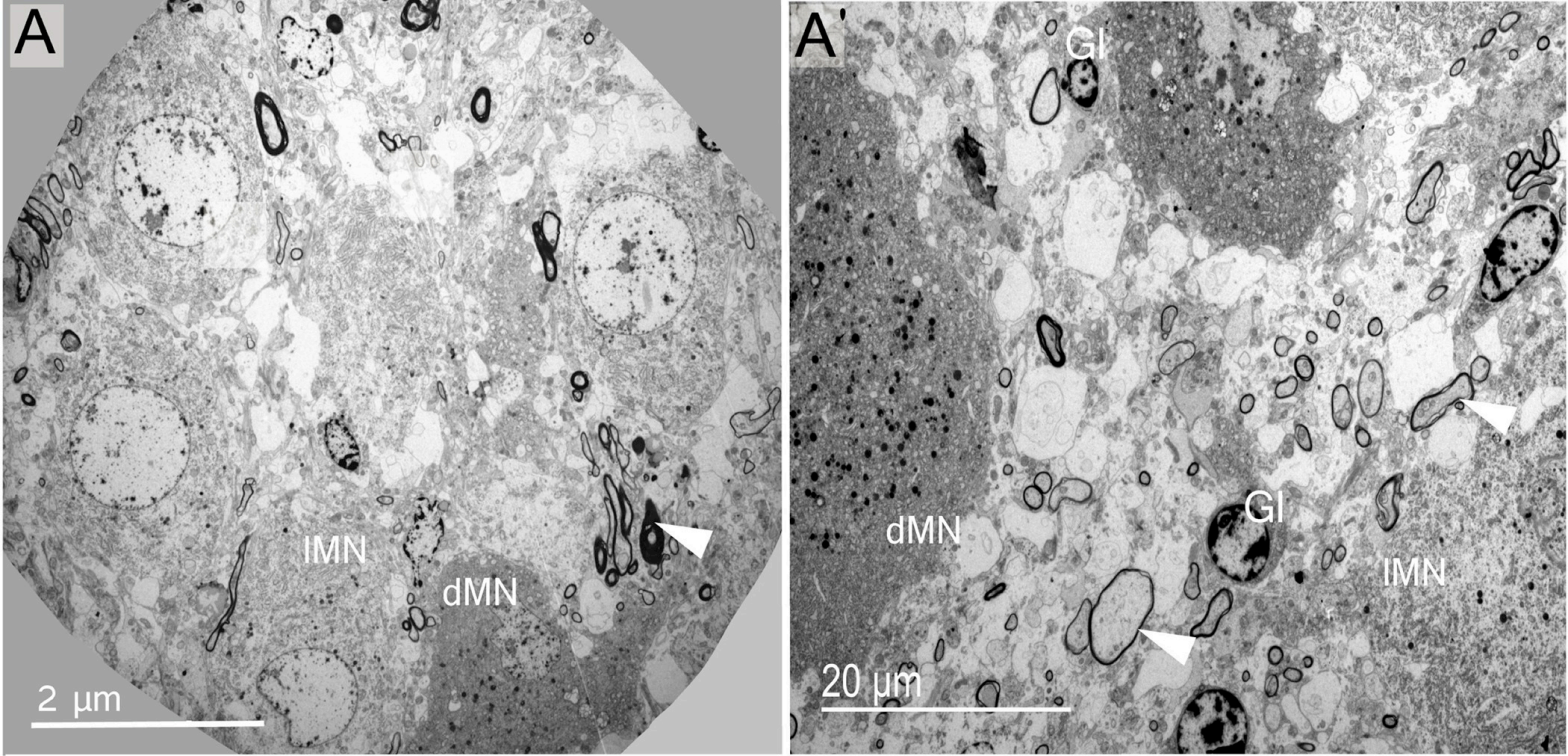
Fine structure of magnocellular neurones. (A, A') Electron microscopic observations of the dromedary SON revealed the presence of two distinct MN phenotypes—light (lMN) and dark (dMN). Light profiles are more abundant than dark. Other important elements found in the SON are myelinated axons (arrowheads). Glial cells (Gl) are observed in different levels of the HNS, and parenchymal glial cells are seen (A').

**Fig 2:**
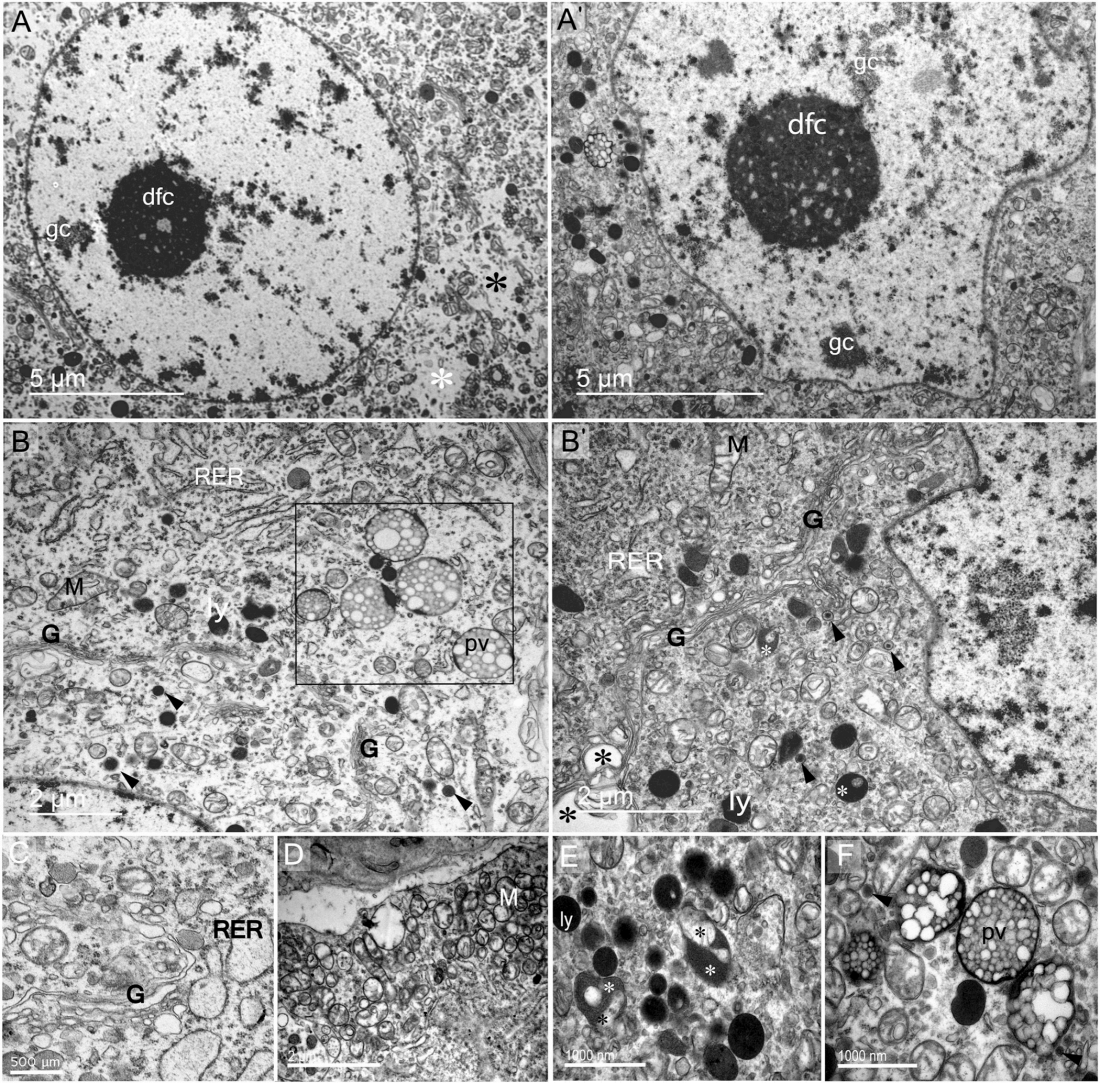
Ultrastructural description of SON magnocellular neurons and their subcellular elements in the somatic zone. Light (lMN) and dark (dMN) magnocellular neurones are morphologically distinct. The nuclei of lMN (A) and dMN (A') are different in shape, respectively rounded and indented. However, both contain dense fibillary condensed (dfc) and granular chromatin (gc). The cytoplasm of the IMN is less loaded (asterisks in A) than that of the dMN. (B-B') Both lMN and dMN showed high activity as reflected by the presence of a well developed machinery of neurosecretion and recycling. Rough endoplasmic reticulum (RER), Golgi apparatus (G), mitochondrion (M), polyvesicular body (pv), lysosomes (ly) and dense core secretory granules (arrowheads; 195.71 nm ± 4.02 and 157.5nm ±3.65) are the main membranous structures found at the subcellular level of MNs. The rough endoplasmic reticulum (RER) was abundant and was present in different degree of dilation (B,C). The rectangle in panel (B) identifies several phagosomes (polyvesicular bodies) with vesicles of heterogeneous size. The Golgi apparatus is scattered as discontinuous (B) or well developed continuous (C). (D) Cluster of mitochondria (M) observed close to plasma membrane. (E) Lysosomes (ly) and autophagosomes (white asterisks), internalizing cytoplasmic membranous structures (black asterisks). (F) Polyvesicular bodies containing heterogeneous vesicles.

**Fig 3:**
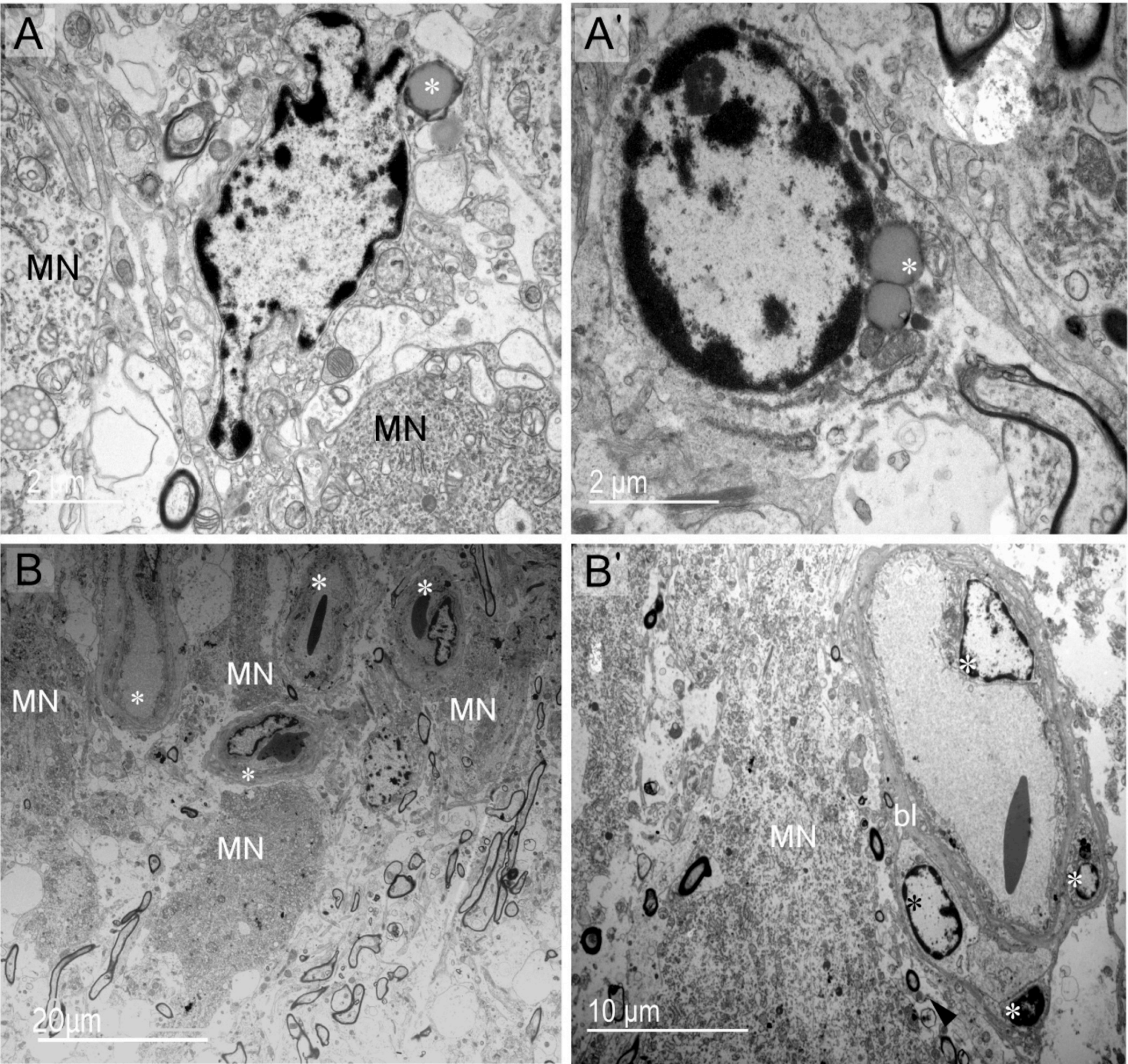
Cell populations and vasculature of supraoptic nucleus at the somatic zone. (A, A') Parenchymal glial cells are distinguished as two groups: cells having smooth rounded nuclei and vesicles similar to lipid droplets (asterisk) (A) and cells with polylobed nuclei containing lipid droplets or endosomes in the cytoplasm (asterisk) (A'). (B)The supraoptic nucleus is richly vascularized. Capillaries (asterisks) are abundant and sometimes close to MNs. (B') Endothelial cells (light asterisks) and pericyte (dark asterisk) of capillaries. bl: basal lamina.

**Fig 4:**
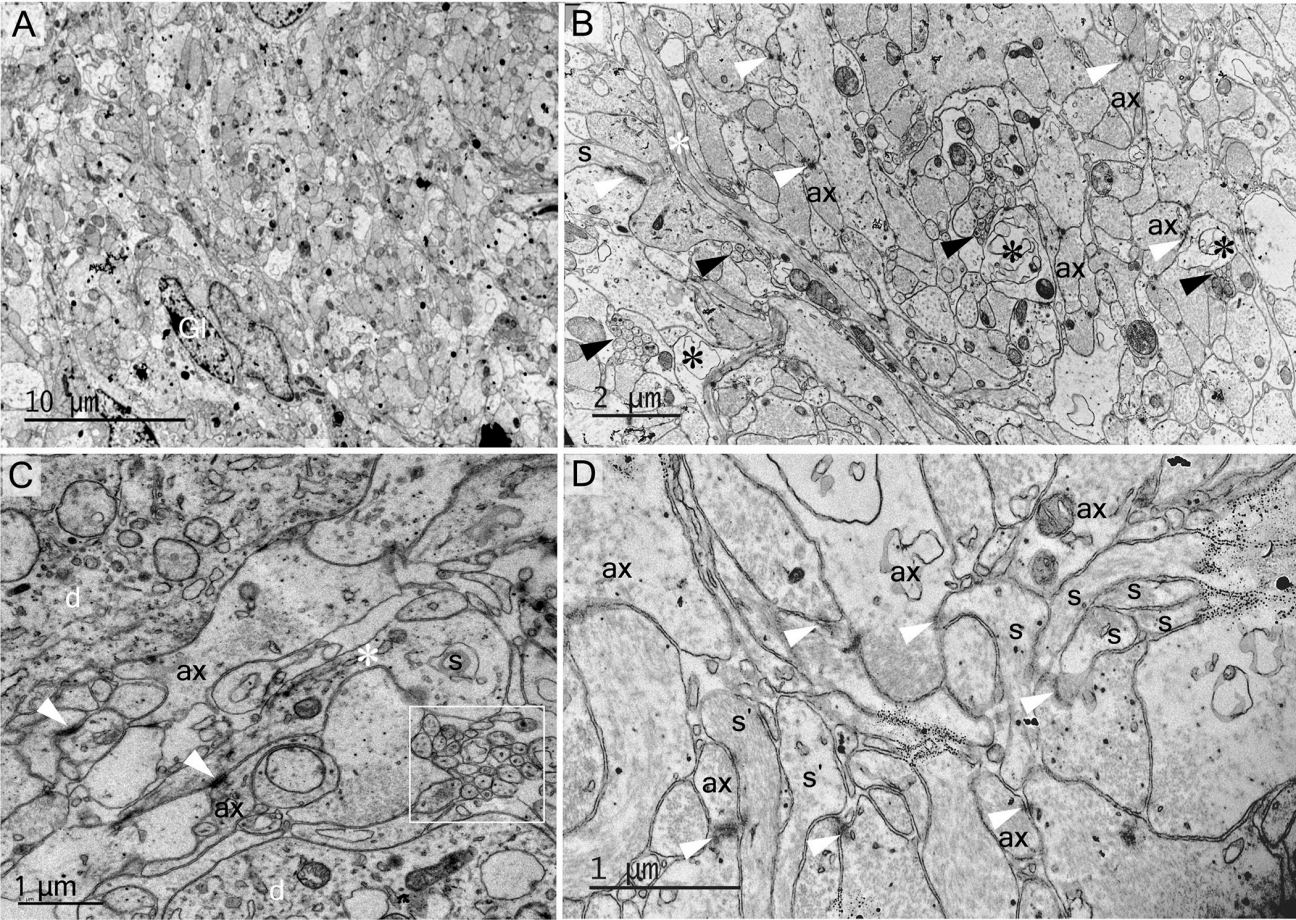
Electron micrographs illustrating the complex organisation of the supraoptic nucleus dendritic zone. (A, B) Dendrites of magnocellular neurones are found with glial cells (Gl) and their processes (light asterisk in B). From these dendrites derive spines (dark arrowheads) that make synaptic contact with axons terminals (ax). Degenerating elements also are observed (dark asterisks in B). (C) A cluster of spines (square) near large dendrites (d). One appears with an apparent neck (asterisk) and head (S) making synaptic contact (arrowheads) with axon terminals (ax). (D) Two clusters of dendritic spines (s and s') organised in bundles and connected to presynaptic elements (arrowheads).

**Fig 5:**
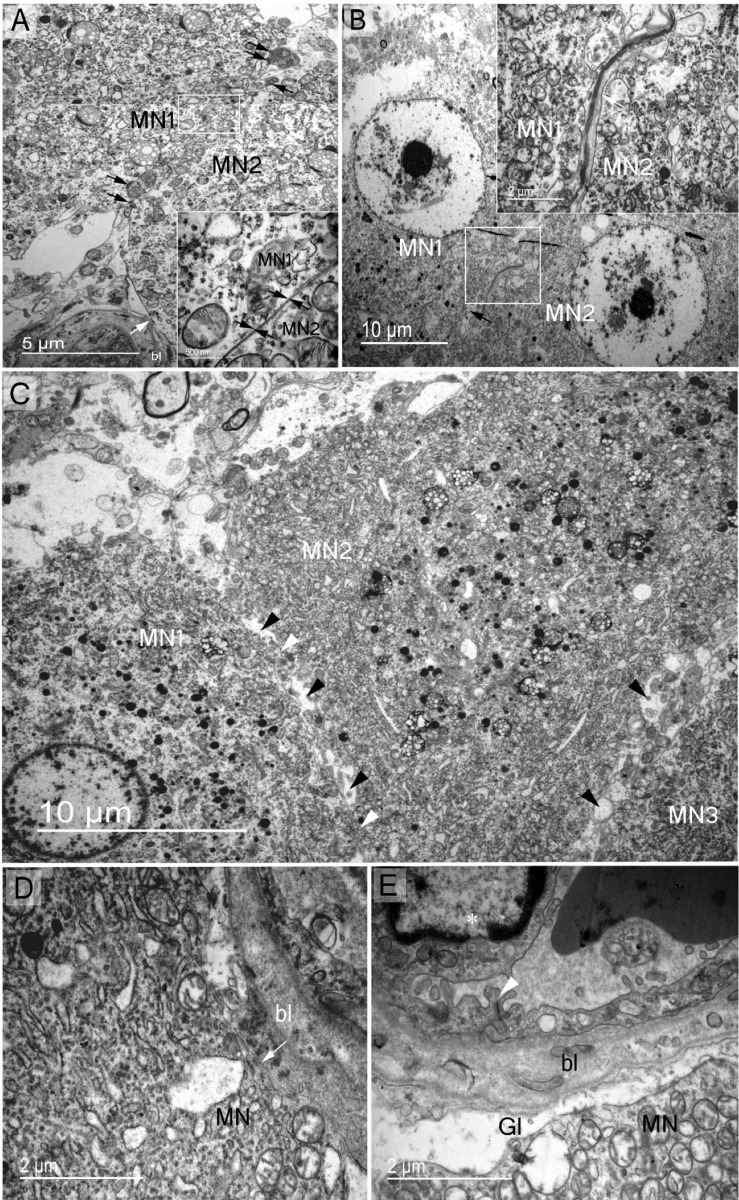
Structural membrane contacts of somatic zone magnocellular perikaryons. Magnocellular neurones establish different types of membrane contact with other supraoptic nucleus elements. (A) Soma-somatic membrane apposition of two adjacent magnocelluar neurones (MN1 and MN2) without intervening neuropil elements; MN1 and MN2 membranes in close contact (arrows in magnified rectangle). Note the presence of active synapses on both MNs (dark arrows). Direct contact of magnocellular neurone membrane with the basal lamina (bl) of a capillary (light arrow). (B) Two adjacent magnocellular neurones (MN1 and MN2) membranes separated by neuropil elements (arrow in the square magnified). (C) Adjacent magnocellular neurones (MN1, MN2 and MN3) with direct soma-soma membrane apposition (white arrowheads between MN1 and MN2) and separated by neuropil elements in several places (dark arrowheads). (D) Direct contact of MN soma membrane with capillary basal lamina (bl) (arrow). (E) Blood barrier in the supraoptic nucleus demonstrating endothelial cells (asterisk) tightly linked (arrowhead) and magnocelluar neurone membrane separated from capillary basal lamina by glial process (Gl).

**Fig 6:**
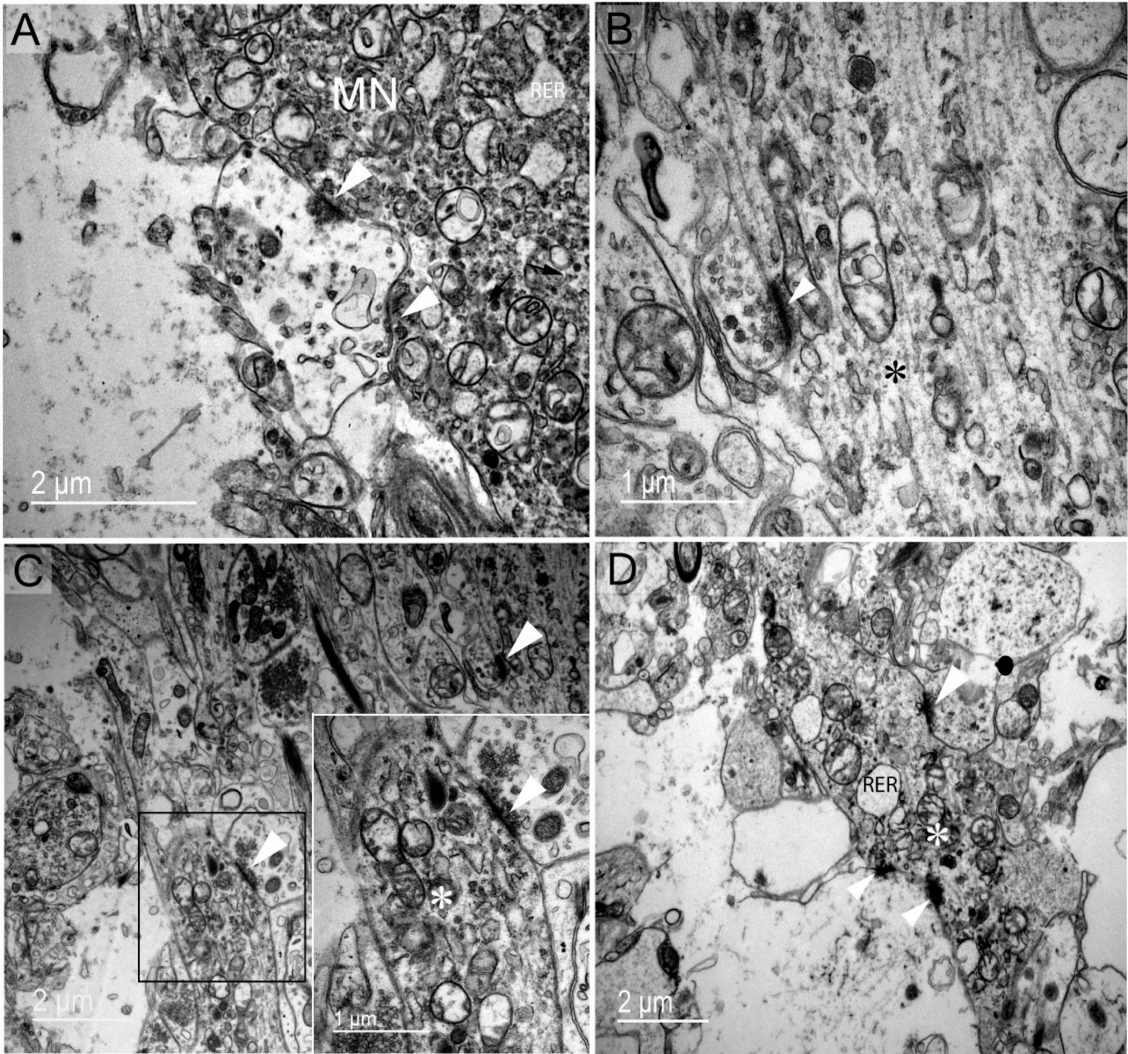
Common synaptic innervations types found in the supraoptic nucleus. (A) Axo-somatic active synapses (arrowheads) were in contact with magnocellular neurones in an active functional stage, according to the cytoplasmic subcellular appearance, especially the dilated rough reticulum endoplasmic. (B) Axo-axonic synapse (arrowhead) (asterisk represents an magnocellular neurone axon). (C) Axo-dendritic synapse (arrowhead) (square magnified in the right) (asterisks represent a dendrite from a magnocellular neurone). (D) In the dendritic zone, multiple axo-dendritic synapses (arrowheads) (asterisk represents a dendrite from a magnocellular neurone) are seen. Note the dilation stage of rough reticulum endoplasmic reticulum (RER).

Electron microscopy was then used to document the detailed cellular morphology of the dromedary supraoptic nucleus somatic and dendritic zones in both winter and summer. In the somatic zone, we have confirmed the presence of dark and light magnocellular neurons (Fig 1) and have described their subcellular elements (Fig 2). In magnocellular neurons we have observed two features of phagosomes both reflecting the phenomena of autophagy and/or heterophagy). Fig 2B and 2F indicate phagosomes enclosing polyvesicular bodies (pv). In Fig 2B' and 2E, phagosomes (light asterisks) seem to be autophagosomes as suggested by the internalization of membranous structures (dark asterisks in E), like a mitochondrion (upper dark asterisk in E). Glial elements (Figs 1 and 3) and the rich vasculature (Fig 3B) have been described. The complex organisation of the supraoptic nucleus dendritic and somatic zones have been documented (respectively, Figs 4 and 5), as have membrane appositions of magnocellular neurons, between dendrites (Fig 4D), and cell bodies (Fig 5) and synaptic innervations (Fig 6).

**Fig 7:**
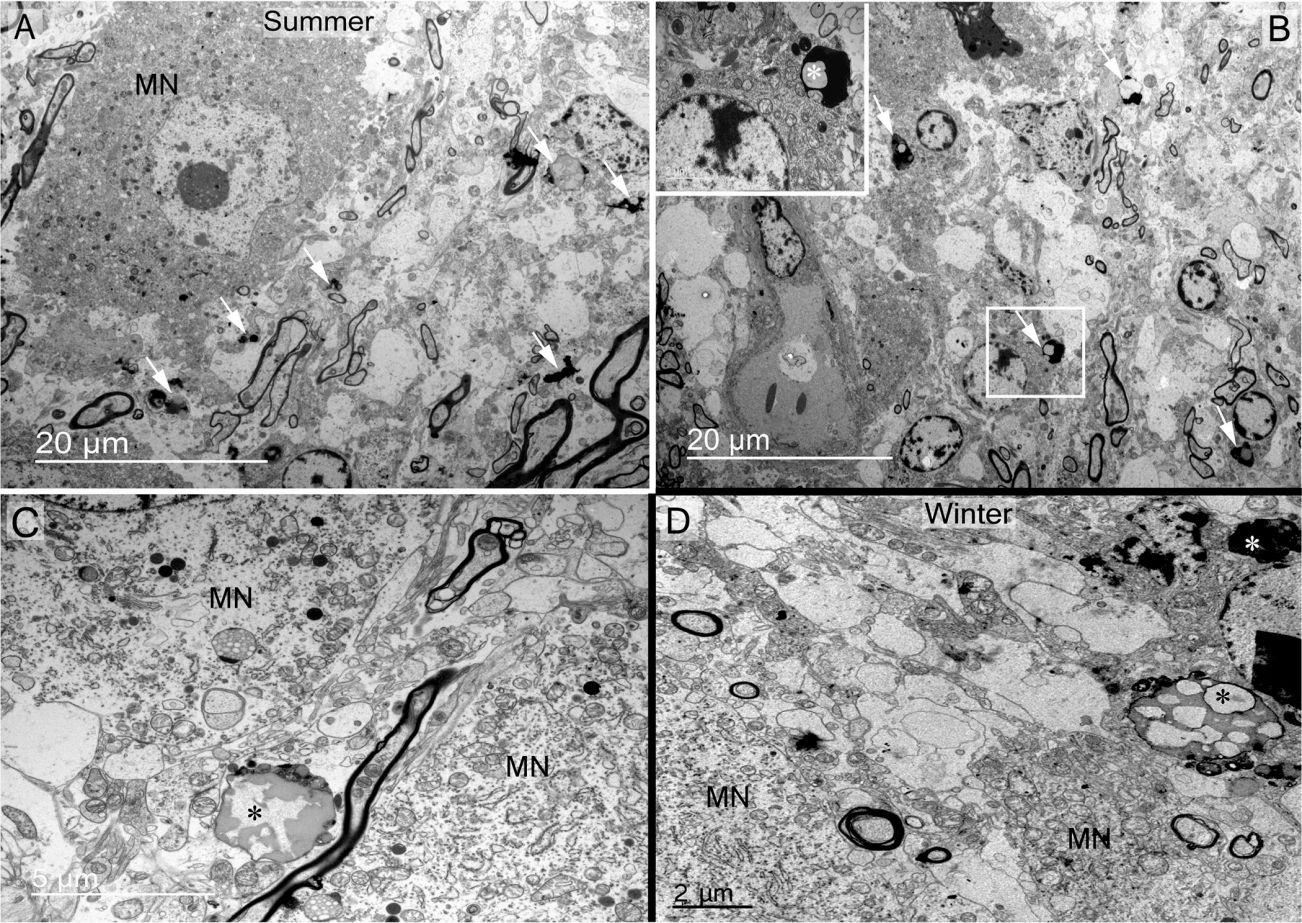
**Debris and degenerating material in somatic zone of the supraoptic nucleus observed in summer (A, B and C) and winter (D) seasons.** (A, B) Debris bodies are found in somatic zone (arrows) localised inside glial cells (arrows in B, and asterisk in rectangle magnified in the top left of B). (C, D) Large degenerative bodies (asterisks) observed between magnocellular neurones close to their membrane.

**Fig 8:**
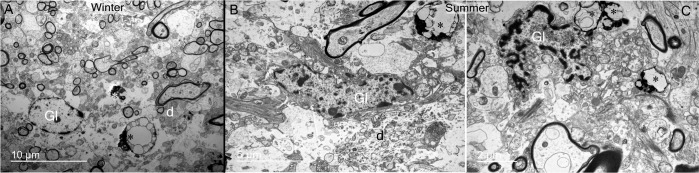
Debris material in supraoptic nucleus dendritic zone observed in summer and winter seasons. (A) Degenerative elements (asterisks) are observed near magnocellular neurone spines and dendrites in both seasons. (B, C) These elements (asterisks) are generally abutted by glial processes (Gl) and engulfed (C). It seems that glial cells (Gl) are attracted to debris zones, and possibly remove them from the neuropil by phagocytosis. Note in B and C that glial cells are engulfing debris (asterisks).

**Fig 9:**
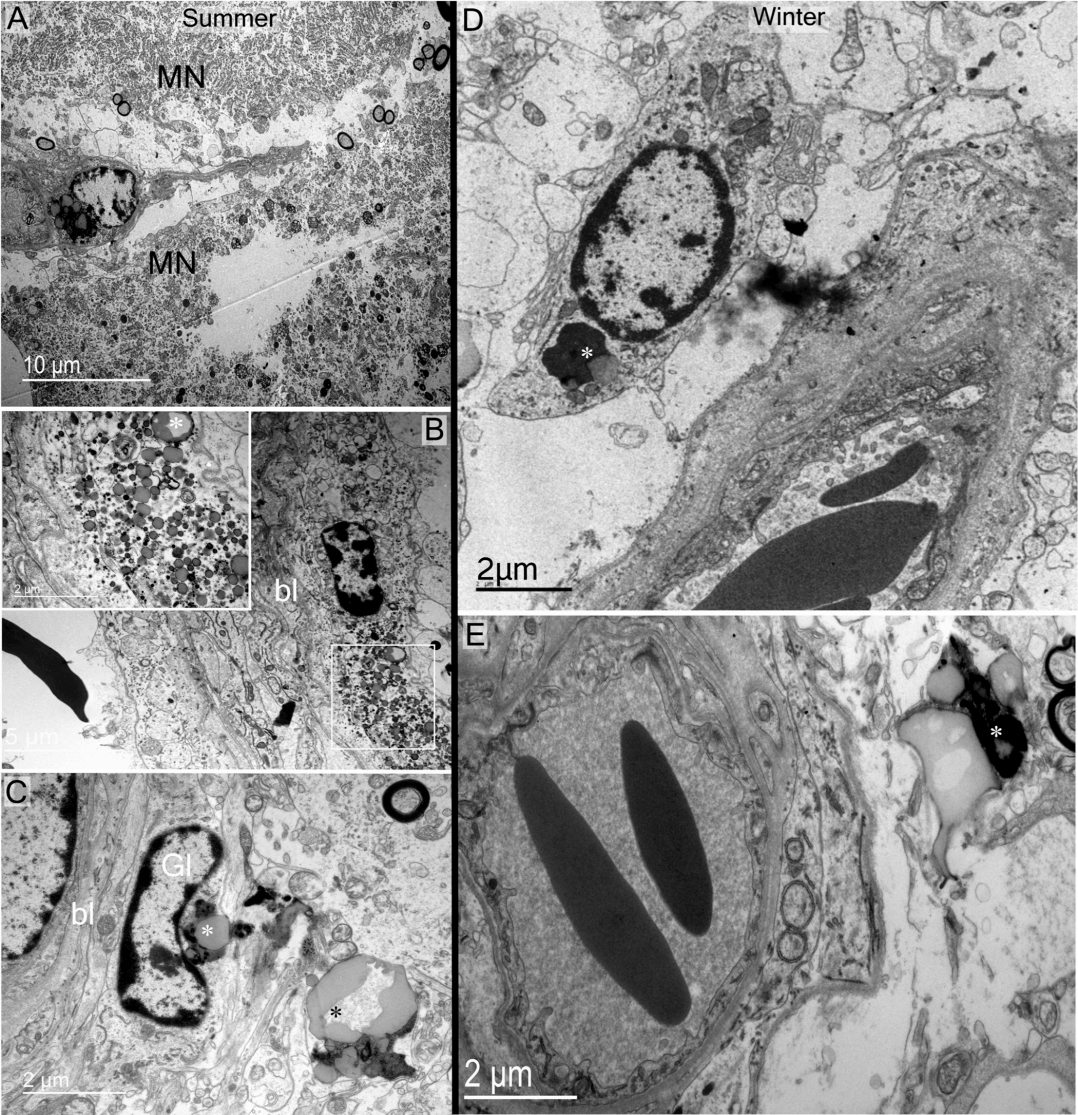
Debris material in perivascular zones of the supraoptic nucleus observed in summer and winter seasons. A-B) Cytoplasmic recycling material is observed in perivascular cells of the somatic zone. (C-E) Debris bodies (asterisks) are also found close to capillaries, engulfed or inside glial cells (Gl). bl: basal lamina; MN: magnocellular neurone.

A striking feature observed was the frequent presence of degenerating material and debris in the somatic zone of the supraoptic nucleus (Fig 7). These bodies are found near or inside glial cells (Fig 7A and 7B) and between magnocellular neurones (Fig 7C and 7D). In the dendritic zone, degenerating material was observed near dendritic spines or dendrites (Fig 8A and 8B) and close to glial phagocytic cells (Fig 8B and 8C). Phagocytosis is indicated by the presence of degenerative cells next to glial cells. As illustrated in Fig 8C, two to three degenerating bodies seem to be in close contact with the membrane and cytoplasm of a glial cell. Recycling material inside capillary cells was also noted (Fig 9A and 9B). Debris bodies are found close to capillaries, free, engulfed or inside glial cells in perivascular zones (Fig 9C–9E).

### Ultrastructural variations between summer and winter seasons

**Fig 10:**
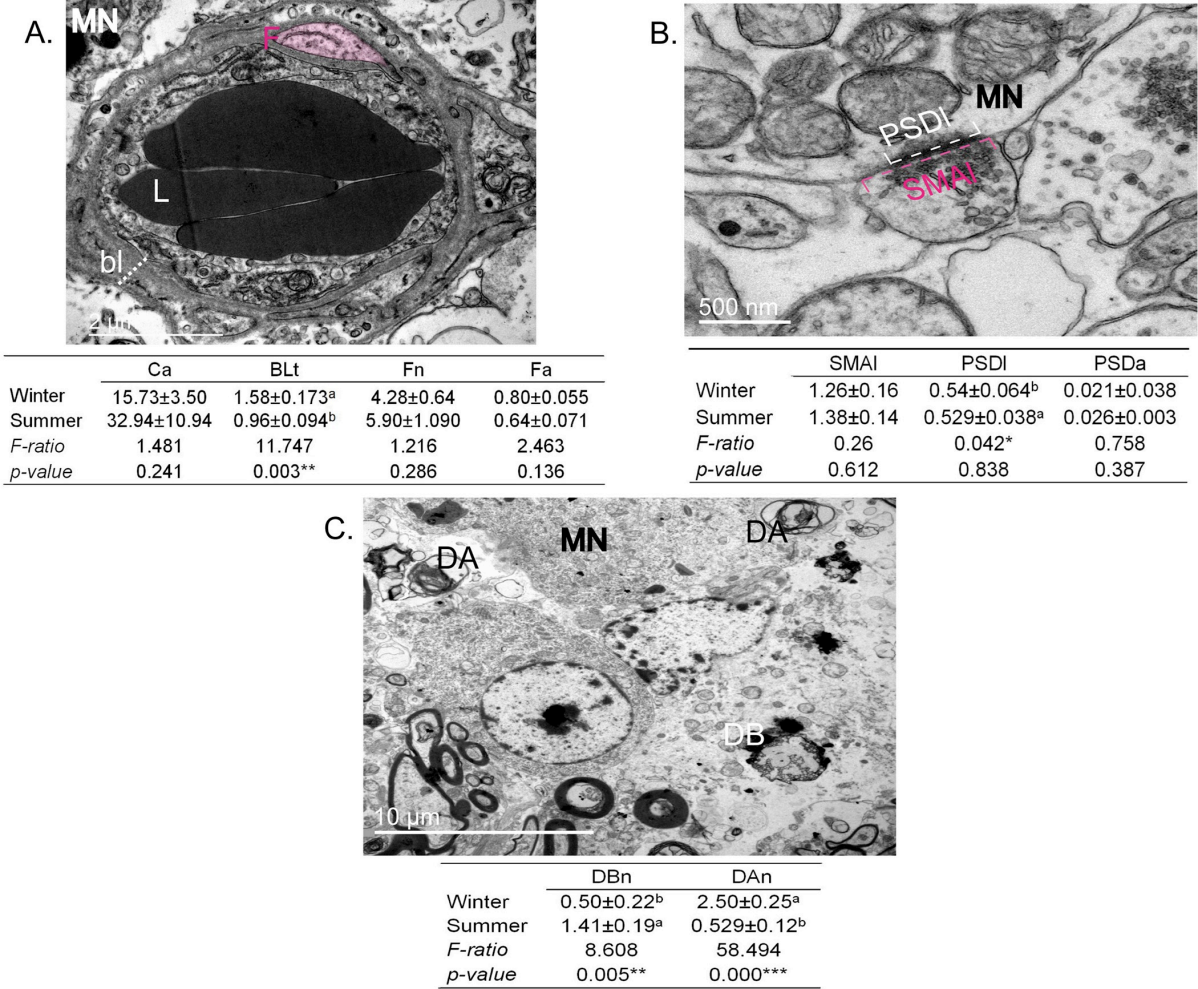
Quantitative analysis of dromedary SON parameters comparing winter and summer. (A) Statistical analysis of capillary parameters (18 capillaries from 3 slices from 3 animals for each season). Ca: area of lumen; BLt: thickness of basal lamina (dashed line); Fn: number of fenestrations (in pink); Fa area of fenestrations. ** Significant seasonal variations (p < 1%). (B) Statistical analysis of synapse parameters (32 synapses from 3 slices from 3 animal for each season). SMAl: synaptic membrane apposition length (dashed lines in pink); PSDl: post-synaptic density length (dashed lines in white); PSDa: post-synaptic density-area (dense area at the side of magnocellular (MN) cell body membrane). * Significant seasonal variations (p < 5%). (C) Statistical analysis of number and kind of debris: DBn: number of degenerating bodies; DAn: number of degenerating axons. ***High significant seasonal variations (p < 0.01%). DA: degenerating body; DA: degenerating axon. F: fenestration; bl: basal lamina; MN: magnocellular neurone; L: lumen.

**Fig 11:**
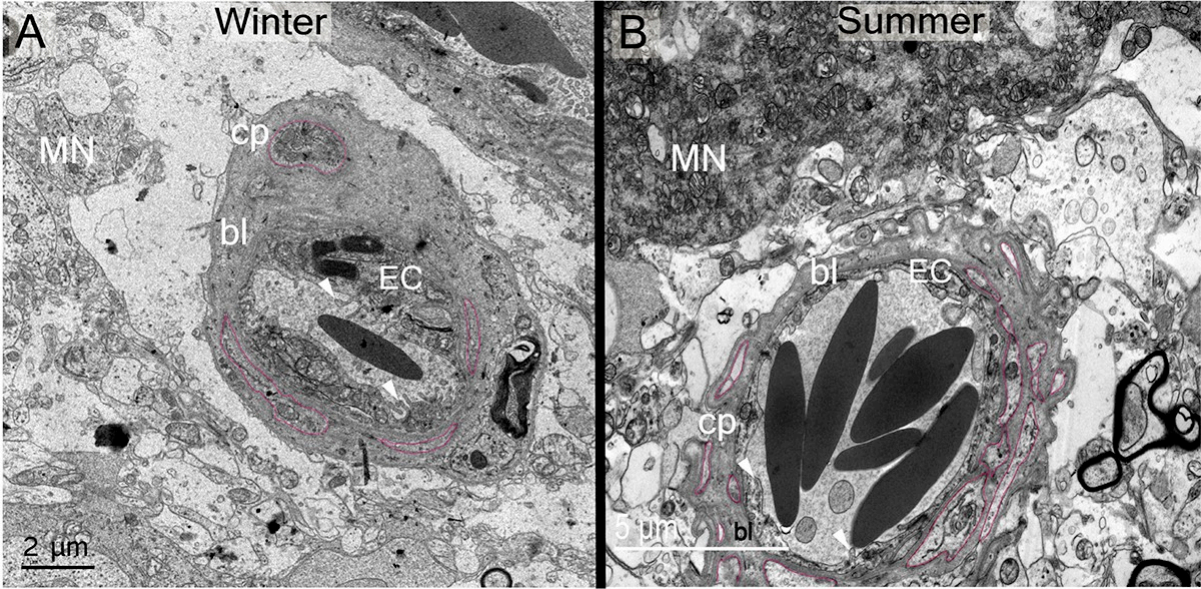
**Electron micrographs showing ultrastructural differences in capillary (cp) basal lamina (bl) between winter (A) and summer (B).** Note the differences in thickness and fenestration (outlined in pink). Note differences between seasons in cytoplasmic expansions (arrowheads) and thickness of endothelial cells (EC).

We performed a statistical analysis of electron microscope images from winter and summer in order to identify differences in supraoptic nucleus morphology. The ultrastructural features analysed were capillaries, synapses and debris (Fig 10). At the level of capillaries, we noticed qualitative differences in the capillary lumen and the thickness and number of fenestrations of basal lamina comparing winter (Fig 11A) and summer (Fig 11B). There were no statistical differences between seasons in capillary-lumen area, number of fenestration and fenestration area (p > 5%). However, basal lamina is significantly thicker in winter compared to the summer season (F = 11.74, p = 0.003, p <5%) (Fig 10A). Analysis of synapse parameters revealed no differences between seasons in post-synaptic density length, synaptic membrane apposition length and post-synaptic density-area (p> 5%) (Fig 10B). The frequency of debris found in our ultrastructural observations was significantly different between winter and summer. In winter compared to summer there are significantly less degenerative bodies (F = 8.608, p = 0.005, p<5%) and significantly more degenerating axons (F = 58.494, p = 0.000, p<0.1%) (Fig 10C).

### The transcriptome of the camel supraoptic nucleus in winter and summer

We used RNAseq to describe the transcriptomes of the camel supraoptic nucleus in both winter and summer (S1 Table). Thus we have identified genes whose expression is altered in this structure according to season (S2 Table). Robust analysis of our data revealed 171 differentially regulated genes (>2 fold difference; p<0.05, n = 2 for each season). Of these, 112 gene transcripts are present at a higher level in winter compared to summer, whilst 59 mRNAs are more prevalent in summer compared to winter.

Only one class of significantly over-represented genes was revealed using the gene ontology tool PANTHER (Protein Analysis THrough Evolutionary Relationships, http://pantherdb.org; [22]) namely neuropeptides (out of 171 genes: expected, 0.23, actual, 6; 25.83 fold enrichment, p = 3.31E-05). Four of these neuropeptide genes are apparently expressed at a higher level in the winter supraoptic nucleus (cerebellin 1, CBLN1; pro-melanin concentrating hormone, PMCH; secretogranin 2, SCG2, Tachykinin 4, TAC4), whilst two are apparently expressed at a higher level in the summer SON (prodynorphin, PDYN; vasopressin, AVP).

### Analysis of neuropeptides in the supraoptic nucleus and neurointermediate lobe in different seasons

**Fig 12:**
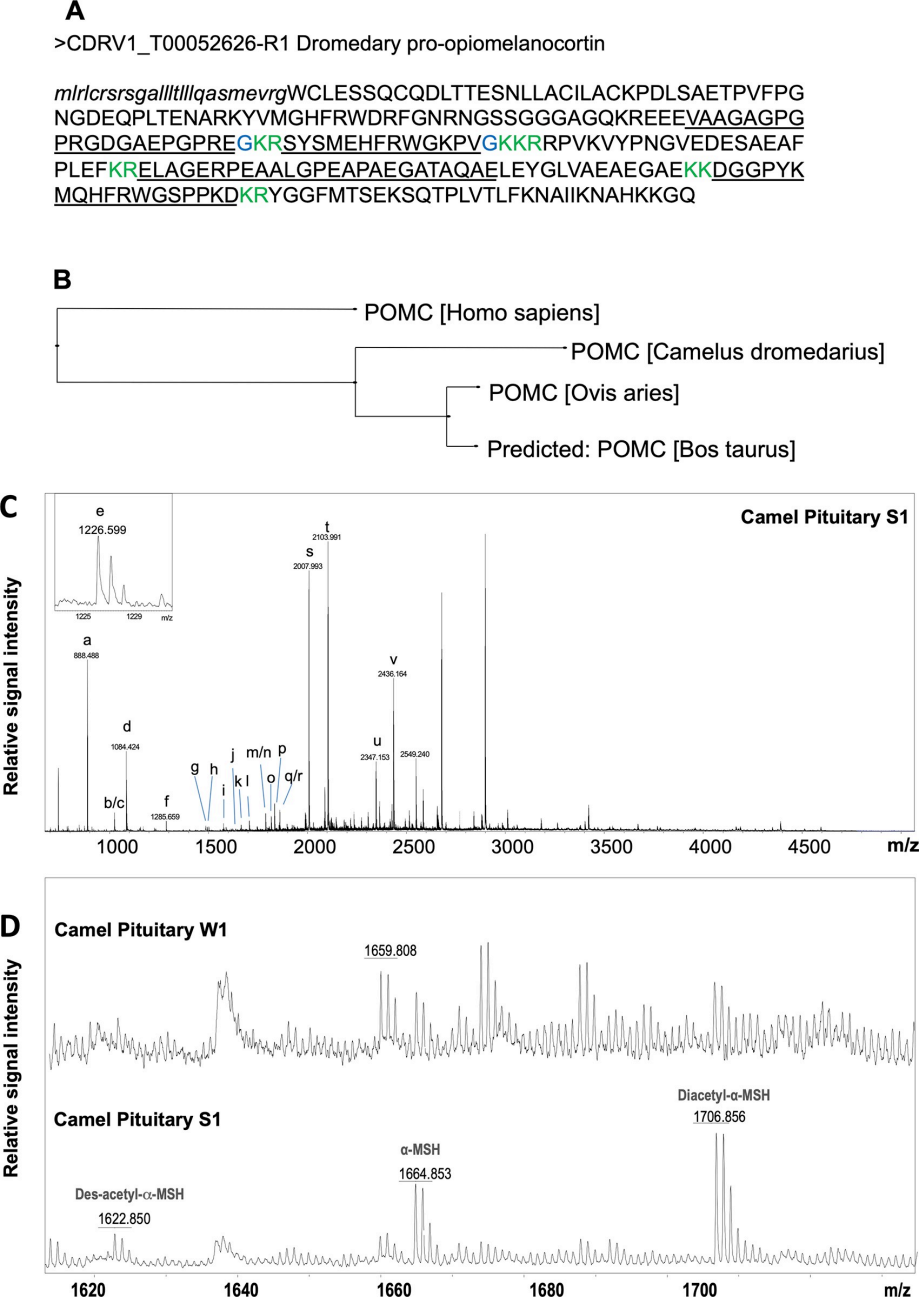
Characterization of a dromedary camel POMC prohormone. (A) Translated protein sequence, predicted signal peptide shown in lowercase letters, confirmed cleavage sites shown in green, confirmed amidation sites shown in blue, peptides detected by MALDI TOF MS underlined. (B) Phylogenetic tree for POMC prohormones from human (P01189), cow (P01190), ship (P01191) and a novel camel sequence that all share 75% identity. This tree was produced using CLUSTALO pairwise alignments and neighbor joining method. Camel POMC shares 89.4% identity with sheep, 88.3% with cow, and 79.3% with human POMC prohormones. Representative MALDI TOF mass spectra of individual camel pituitary extracts. (C) Full mass range spectrum from a summer sample, peaks matching the masses of predicted peptides are labelled (Table 1); (D) Zoom-in view showing differential detection of neurotensin, melanotropin alpha, α-MSH, and its post-translationally modified forms depending on the season. Labels: W, winter; S, summer.

We used matrix-assisted laser desorption/ionization time-of-flight mass spectrometry (MALDI-TOF MS) to assess the presence of peptides in extracts of the camel supraoptic nucleus and neurointermediate lobe in both winter and summer [23,24]. Our transcriptome data identified a longer proopiomelanocortin (POMC) protein than the predicted NCBI sequence for dromedary POMC (Fig 12A and 12B). In the neurointermediate lobe, using peptide mass fingerprinting approach [25], we identified 10 peptides from this novel dromedary POMC prohormone (Fig 12C) and 3 peptides from the AVP prohormone (Table 1). All known forms of α-melanocyte stimulating hormone (α-MSH) (des-acetylated, mono-acetylated, and di-acetylated), were detected in the neurointermediate lobe in the summer samples. In winter samples, signal matching predicted masses of α-MSH peptides was below signal-to-noise ratio cut off for confident peak detection and assignment (Fig 12D). Select peptides matching predicted masses by peptide mass fingerprinting as well as AVP were confirmed by tandem mass spectrometry in a follow up peptidomic analysis (S5 Fig).

**Table 1:** Peptide mass fingerprint assignment of peptides in the camel neurointermediate lobe extracts as determined by MALDI-TOF MS.

Label	Sequence	(M+H)theor	(M+H)exp*	Accuracy	Prohormone
a	FRWGKPVa	888.52	888.52	3	POMC
b	ac-GDGAEPGPREa	1025.47	1025.63	-159	POMC
c	HFRWGKPVa	1025.58		-47	POMC
d	cYFQNcPRGa	1084.45	1084.54	-85	AVP
e	HFRWGSPPKD	1226.61	1226.62	-9	POMC
f	MEHFRWGKPVa	1285.66	1285.67	-4	POMC
g	MQHFRWGSPPKD	1485.71	1485.74	-21	POMC
h	AGAPEPAEHAQPGVY	1493.70	1493.74	-24	AVP
i	GAGPGPRGDGAEPGPREa	1575.76	1575.8	-22	POMC
j	des-ac-SYSMEHFRWGKPVa	1622.79	1622.82	-18	POMC
k	ac-SYSMEHFRWGKPVa	1664.80	1664.83	-16	POMC
l	di-ac-YSMEHFRWGKPVa	1706.82	1706.82	0	POMC
m	pGlu-LAGERPEAALGPEAPAE	1788.88	1788.88	0	POMC
n	RPVKVYPNGVEDESAE	1788.88		0	POMC
o	VAAGAGPGPRGDGAEPGPREa	1816.91	1816.91	0	POMC
p	VQLAGAPEPAEHAQPGVY	1833.92	1833.92	0	AVP
q	DGGPYKMQHFRWGSPP	1859.87	1859.92	-26	POMC
r	RPVKVYPNGVEDESAEA	1859.92		-2	POMC
s	RPVKVYPNGVEDESAEAF	2006.98	2006.95	17	POMC
t	DGGPYKMQHFRWGSPPKD	2102.99	2102.96	14	POMC
u	RPVKVYPNGVEDESAEAFPLE	2346.16	2346.13	14	POMC
v	ELAGERPEAALGPEAPAEGATAQAE	2435.17	2435.14	13	POMC

Monoisotopic theoretical mass computed from the amino acid sequence of protonated molecular ion (M+H); *Mean (M+H) computed from all neurointermediate lobe samples where predicted mass has been experimentally detected; Accuracy of detection is represented as parts per million, ppm; PTMs are italicized lower case letters: a-amidation, ac-acetylation, c-half disulfide bond, pGlu-pyroglutamic acid. POMC: proopiomelanocortin. AVP: arginine vasopressin; Peptides labelled m/n and q/r could not be distinguished and thus both sequences are given.

Assignment of peptides by mass match in MALDI-TOF MS spectra from supraoptic nucleus samples was not possible except for AVP (*m/z* 1084.5) The assignment of AVP in supraoptic nucleus was possible because the same *m/z* was detected in the neurointermediate lobe samples (S6A Fig) and identified as AVP in follow-up liquid chromatography-mass spectrometry/mass spectrometry analysis of pituitary extract (S5A Fig). We then used molecular formula modelling to analyse isotopic pattern of *m/z* 1084.5 detected by MALDI TOF MS in supraoptic nucleus and found it matched the theoretical isotopic pattern of AVP (S6B Fig).

**Fig 13:**
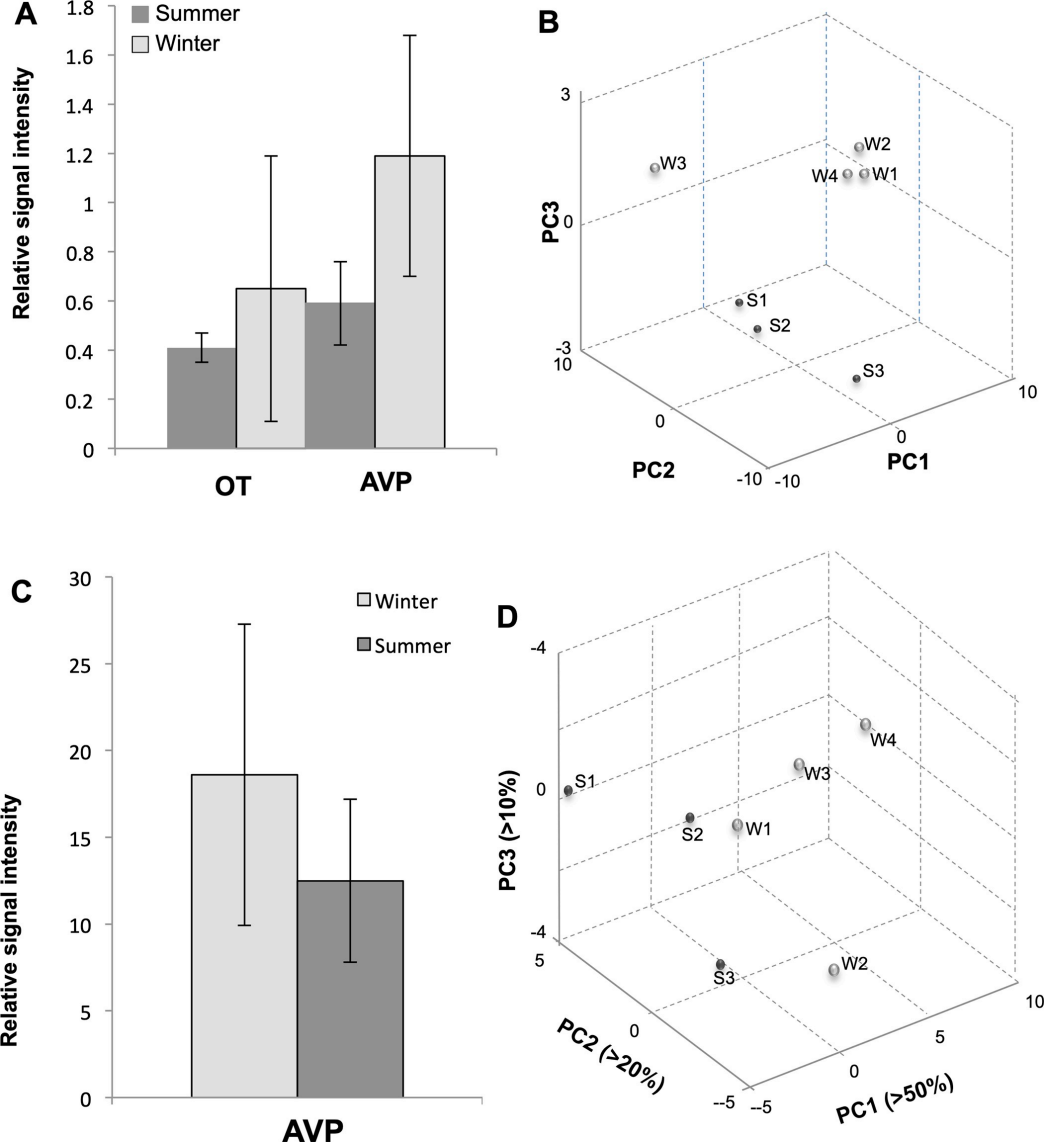
Seasonal peptide level change in camel supraoptic nucleus and neurointermediate lobe. (A) Signal intensity changes of select peptide ions in camel supraoptic nucleus at different seasons. Labels: AVP, arginine vasopressin; OT, oxytocin. (B) Principal component analysis plot for the first three principal components (PC) allows discrimination between seasonal supraoptic nucleus samples based on peptide profile change, S1-S3, summer samples; W1-W4, winter samples, W3 samples is an incorrectly isolated sample. (C) Signal intensity changes of AVP ions in camel neurointermediate lobe at different seasons. Labels: AVP, arginine vasopressin. (D) Principal component analysis plot for the first three principal components (PCs) allows discrimination between seasonal neurointermediate lobe samples based on peptide profile change. S1-S3, summer samples; W1-W4, winter samples.

Comparison of the entire peptide profiles in supraoptic nucleus and neurointermediate lobe raw extracts by principle component analysis [26,27] allowed us to classify both sample types by season (Fig 13). We found that, 4 and 5 principal components (PCs) were required to explain 95% of variance in the supraoptic nucleus and neurointermediate lobe spectra datasets, respectfully. Segregation of spectra by season achieved along PC2 and PC3 in SON, although PC1 accounted for more than 40% of variance. In the neurointermediate lobe, PC1 accounted for more than 50% of variance and effectively segregated the spectra by season. Due to few samples available for mass spectrometry and resulted low number of measurement per sample group (4 x2technical replicate = 8 for winter and 3x2technical replicate = 6 for summer), the sample size was insufficient to determine significance of individual peptide contribution to seasonal differences (detected by principal component analysis) using univariate statistics. Nevertheless, trends in seasonal level changes were inferred for AVP and OT by comparing respective ion signals measured by MALDI TOF MS in supraoptic nucleus and neurointermediate lobe extracts from summer and winter samples (Fig 13A and 13C). The level of difference is expressed as signal fold change calculated from normalised average ion intensity in the season group spectra (Tables 2 and 3).

**Table 2:** Peptide signal fold change in camel SON extracts at different seasons as measured by MALDI-TOF MS.

Peptide	(M+H)_exp_	Intensity* Summer	Intensity* Winter	Ratio Summer/Winter
OT	1007.6	0.41±0.06	0.65±0.54	0.6
VP	1084.8	0.59±0.17	1.19±0.49	0.5

*Normalized (to total ion count) mean relative signal intensity for centroid peaks matching masses of vasopressin (AVP), oxytocin (OT) in the camel SON samples from different seasons (winter n = 4, summer n = 3)

**Table 3:** AVP peptide signal fold change in camel neurointermediate lobe extracts at different seasons as measured by MALDI-TOF MS.

Peptide	(M+H)_exp_	Intensity* Summer	Intensity* Winter	Ratio Summer/Winter
VP	1084.6	18.6±8.7	12.19±4.7	0.7

*Normalized (to total ion count) mean relative signal intensity for centroid peaks matching mass of vasopressin (AVP) in the camel neurointermediate lobe extract samples from different seasons (winter n = 4, summer n = 3), as measured by MALDI-TOF MS.

### Peptide characterization in the supraoptic nucleus by tandem mass spectrometry

Peptides were sequenced using liquid chromatography-tandem mass spectrometry and identified via automatic de novo spectra interpretation followed by de novo tag search [28] against the NCBI protein database for *Camelus dromedarius*; results were filtered at 1% FDR for peptide-spectrum matches. A total of 301 proteins supported by 918 peptides were identified from pooled winter SON sample, while 277 proteins and 988 peptides were identified from summer SON (S3 Table). Additional peptides were identified when search was repeated against custom annotated database based on our RNAseq data. The presence of the PMCH hormone by detection of a single peptide, Neuropeptide-glutamic acid-isoleucine (EIGDEENSAKFPI-amide), only in winter SON. SCG1- and SCG2- derived peptides were detected in both seasons, but more peptides identified in winter than summer for either of the proteins. Peptides from SCG3 were detected only in winter SON samples. We found evidence of alternative splicing of the tachykinin precursor 1 in dromedary between seasons (S7 Fig and S4 Table). In summer we detected neurokinin A (isoforms 2,4, and 6) as well as peptide DADSSVEKQVALLKALYGLGQISHKMAYE confirming prohormone variant without neurokinin A (dromedary isoforms 3 and 7), while in winter SON we detected peptides supporting prohormone variants with neurokinin A (isoforms 2, 4, 6). Substance P was detected only in winter samples. Thus, MS data supported some of our transcriptomics results.

### Seasonal effects on circulating hormone levels

**Fig 14:**
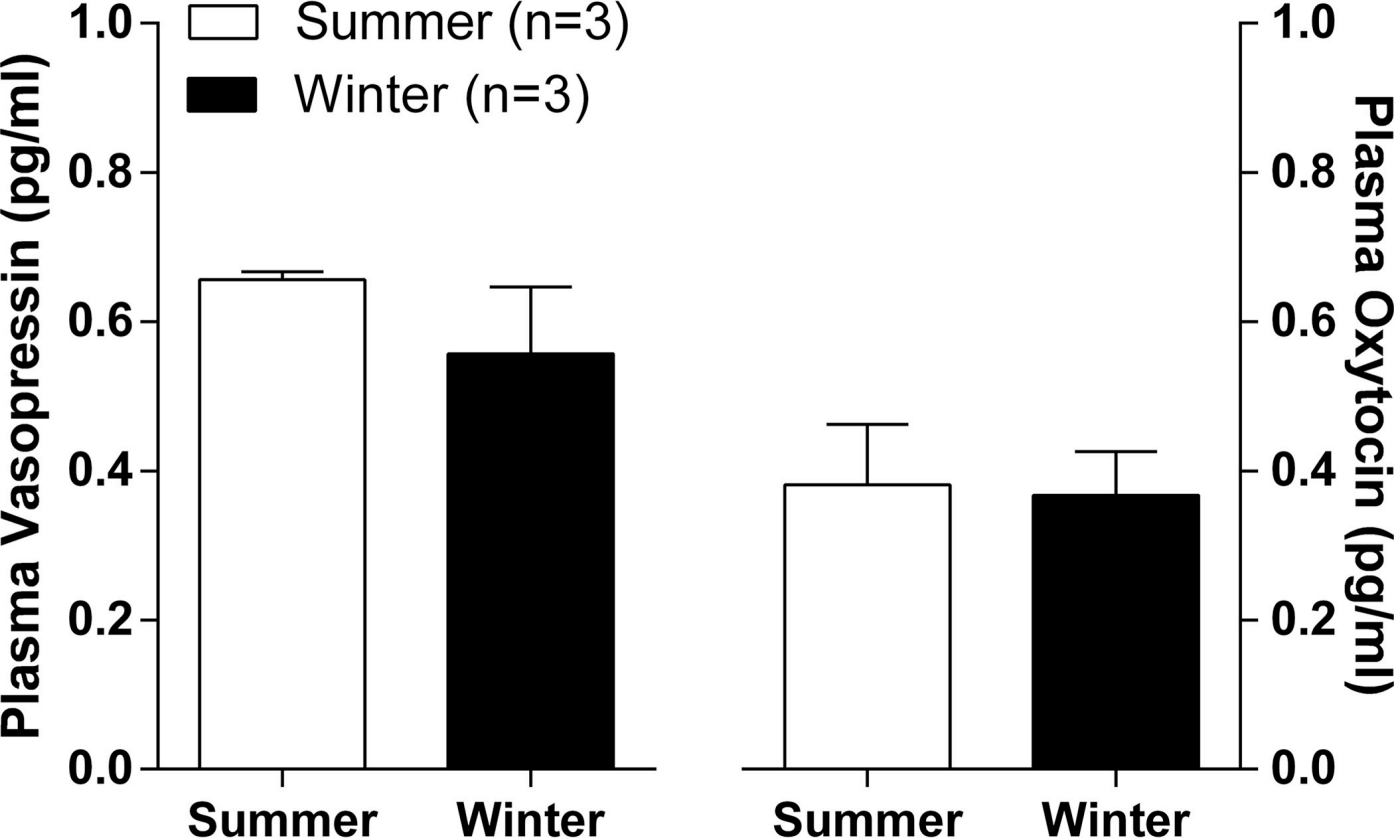
Circulating plasma AVP and OT levels are unchanged with season in hydrated males dromedary camels as determined by radioimmunoassay.

As measured by radioimmunoassay [29], comparing winter and summer, no significant differences were observed in plasma levels of AVP or OT (Fig 14).

## Discussion

The supraoptic nucleus is the biosynthetic core of the HNS, the specialized part of the central nervous system devoted to centrally overseeing the brain body-dialogue that ensures the regulation of water balance. In this study, we have integrated genomic, transcriptomic, proteomic and morphological approaches to better understand the role of the supraoptic nucleus in the extraordinary ability of the dromedary camel to survive in the hot, arid conditions of the desert summer.

The dromedary supraoptic nucleus starts rostrally with a group of neurones in a dorsal position to the optic chiasm and then lengthening dorsolaterally. This is similar to what has been observed in small desert mammals such as the *Meriones shawi* [30]. AVP and OT neurons were found intermingled throughout the nucleus, which is consistent with an earlier work on the identification of camel HNS secretory products [31].

It is well established in the rat that the supraoptic nucleus is a dynamic system, with its elements undergoing reversible morphological plasticity in response to stimuli [32,33]. We have previously used electron microscopy to show that the morphology of camel posterior pituitary changes according to season [20]. The storage of neuropeptides is very marked in summer and is associated with autophagic and phagocytic phenomena, suggesting seasonal adaptation to anticipate any situation that would cause dehydration.

Examination of the fine structure of the dromedary supraoptic nucleus by electron microscopy revealed the presence of two phenotypic magnocellular perikarya, identified as light and dark (Figs 1 and 2). It is possible that the dark and light neurosecretory elements previously described in the camel posterior pituitary [20] could be derived from the dark and light perikaryons observed in supraoptic nucleus. Pelevin and Zelenskaya [34] also reported the presence of the two types of magnocellular neurone in rats, and suggested that the difference is a consequence of high or low activity of light or dark magnocellular neurones respectively. However, in the same species, other researchers obtained the dark type after intraperitoneal injection of neuroleptic drugs, suggesting that these are perhaps exhausted neurosecretory neurons [35]. We suggest that the two types of magnocellular neurones in dromedary supraoptic nucleus may have a distinct neurosecretory products due to differences in ultrastructural organization and the abundance of light cell bodies and light terminals compared to dark types. Magnocellular neurone biosynthetic activity is evidenced by the presence of abundant membranous structures, numerous mitochondria, dilated endoplasmic reticulum and well developed Golgi apparatus.

The dromedary supraoptic nucleus showed membrane appositions characterised by the formation of bundled dendrites in the dendritic zone (Fig 4) and membrane appositions between neighbouring MNs in the somatic zone (Fig 5). This organisation may be responsible for the coordination of information on postsynaptic elements [36]. The magnocellular neurones of the camel supraoptic nucleus receive rich and diverse synaptic inputs. Axo-somatic, axo-axonic and axo-dendritic types were all observed (Fig 6). Previous studies have identified inputs from the medial preoptic nucleus to AVP magnocellular neurones in sheep [37]. The medial preoptic area constitutes an important structure implicated in osmoregulation and is believed to be a major source of supraoptic nucleus input in the rat [38]. In addition, magnocellular neurones of the supraoptic nucleus receive direct inputs from the subfornical organ and the organum vasculosum of the lamina terminalis, or indirectly from these structures via the medial preoptic area [39,40].

Similar to the rat [41], the dromedary supraoptic nucleus is highly vascularized with a dense capillary network, Ultrastructural analysis (Fig 3B and 3B') demonstrated a close structural relationship of magnocellular neurones with the capillary basement membrane. Magnocellular neurones are thought to act as cerebral osmoreceptors [42], and Mason [43] suggested that magnocellular neurones are themselves directly osmosensitive and are part of an osmoreceptive complex. Gross *et al*. [44] and Muchlinski *et al*. [45] suggested that microvessel density and distribution makes the nucleus sensitive to small perturbations in plasma osmolality, and may facilitate the access of stimulating or inhibiting plasma factors to magnocellular neurones. They could also enable the supply of circulating glucose needed for sustaining a high metabolic activity [46]. We observed differences in the vascularisation of the supraoptic nucleus according to season. Particularly, in winter, the blood capillary basal lamina is thicker compared to vessels in summer (Fig 11). In perivascular zones of the central nervous system, the basal lamina is an important element of the vascular unit, and is secreted by endothelial, pericytes and astrocytes cells [47]. The basal lamina is mainly composed of fibrous proteins (laminins and collagen IV isoforms) and proteoglycanes (nidogen and heparin sulfate) that separates the endothelial cells from pericytes, glial cells and neurons and plays a role in maintaining the cells of the vascular unit [48]. The vascular basal lamina contributes to vessel development and formation [49], and functions as physical barrier surrounding the abluminal surface of endothelial cells, contributing to the maintenance of the blood-brain barrier [50]. Thickening of the dromedary supraoptic nucleus basal lamina in winter, as compared to summer, could have effects on endothelial barrier function, perhaps by protecting neurones from fluctuations in plasma composition [51], or by promoting vascular stability [52]. In this regard, our transcriptome data revealed an interesting elevation in the expression of GPR124 transcripts in winter compared to summer. GPR124, an orphan member of the adhesion G protein coupled receptor family, is essential for central nervous system angiogenesis, and for the formation of the blood-brain barrier [53]. The expression of the KDR (kinase insert domain receptor) gene, that encodes a receptor for vascular endothelial growth factors (VEGFs) receptor 2 protein, is decreased in winter compared to summer. In the rat, local secretion of VEGFA in the supraoptic nucleus has been implicated in the angiogenesis induced by hyperosmotic cues [54].

A striking feature found in the parenchyma of dromedary supraoptic nucleus was the frequent presence of debris and degenerative elements, scattered between the magnocellular neurones somata and their dendrites and in perivascular zones. Displaying the appearance of degenerating neural elements and dead cells, these structures were also frequently observed in the cytoplasm of glial cells with polylobed nuclei of the microglial type. Interestingly, these parameters change significantly with season. The number of degenerating residual bodies is significantly higher in summer compared to winter, whereas in winter, significantly more degenerating axons are present compared to summer. These data suggest that the camel supraoptic nucleus is under intense stimulation in summer, with reduced astrocytic coverage allowing increased synaptic input. This is reversed in winter, as evidenced by the number of degenerating inputs [55], perhaps indicating reduced excitatory inputs. Similarly, we have previously reported high phagocytic activity in the summer neural lobe [20],

Different types of microglia have been previously identified using specific markers in rat supraoptic nucleus [56]. After stimulation of rat supraoptic nucleus by salt-loading, Ayoub and Salm [57] observed increased morphological diversity of microglia, suggesting that these cells may be involved in regulating peptides and other substances released in the activated nucleus. A perivascular population of microglia has been reported [58], which could be implicated in the degradation of basal lamina proteins [59]. Microglia may also be a source of cytokines that influence magnocellular neurones [60] and promote dendritic release of neuropeptides [61].

Our transcriptomic data supports the electron microscope observations, suggesting increased microglial activation in summer compared to winter. Out of the 59 genes that we identified as being significantly expressed at a higher level in summer compared to winter, 7 have been implicated in the activation of microglia (tumor necrosis factor superfamily member 18, TNFSF18, [62]; C-C motif chemokine receptor 5, CCR5, [63]; glycoprotein non-metastatic melanoma B, GPNMB, [64]; heparin binding EGF like growth factor, HBEGF, [65]; signal regulatory protein β-1, SIRPB1, [66]; annexin-1, ANXA1, [67]), or are markers of activated microglia (CD44, [68]).

Gene ontology mining of our transcriptome data revealed only a single over-represented class of proteins, namely neuropeptides. Four of these neuropeptides are expressed at a higher level in winter (CBLN1, PMCH, SCG2, TAC4), whilst two are expressed at a higher level in summer (PDYN, AVP). An increased expression of PDYN and AVP would be expected during a period of osmotic stress, and this is indeed what is observed in the dehydrated rat [69].

We were interested to see if any of the transcripts regulated by season in the dromedary supraoptic nucleus were also regulated by chronic dehydration in the rat supraoptic nucleus, as revealed by our transcriptome analysis [70]. Only 5 common genes were revealed: DAZ associated protein 1 (DAZAP1; a germ cell RNA binding protein, expression increased with dehydration in the rat, increased in dromedary in winter compared to summer), Fibronectin 1 (FN1; involved in cell migration and adhesion, decreased with dehydration in the rat, decreased in dromedary in winter compared to summer), prodynorphin (PDYN, increased with dehydration in the rat, decreased in dromedary in winter compared to summer, see below), retinoic acid receptor responder 2 (RARREST2; Adipocyte-secreted protein, decreased with dehydration in the rat, decreased in dromedary in winter compared to summer) and secretogranin II (SCG2; packaging and sorting of peptide hormones into secretory vesicles, increased with dehydration in the rat, increased in dromedary in winter compared to summer, see below).

We then embarked on a more in-depth peptide analysis of the dromedary supraoptic nucleus and neurointermediate lobe that, necessarily coupled with our genomic and transcriptomic data, has revealed novel insights. Our data also suggest that seasonal changes in select supraoptic nucleus mRNA content results in corresponding changes in peptide content. Thus, we found elevated levels of PMCH, PDYN and SCG2 mRNAs in winter supraoptic nucleus compared to summer. Similarly, these peptides were detected only in winter samples. However, whilst we saw elevated AVP mRNA expression in the summer supraoptic nucleus compared to winter, this was accompanied by a decrease in AVP peptide content in both supraoptic nucleus and neurointermediate lobe. That said, decreased HNS AVP content is entirely consistent with an increase in AVP gene expression under dehydrating conditions. We suggest that AVP gene expression is increased in summer as a consequence of dehydration, or in preparation for the likely prospect of dehydration. However, as is observed in the rat [15], periods of chronic osmotic stress result in an increase in the axonal transport of AVP from the site of synthesis in the cell bodies of the supraoptic nucleus to the site of storage and release in the axon terminals of the posterior pituitary [15,71], resulting in a decrease in steady-state peptide levels within the supraoptic nucleus. Similarly, increased axonal release as a result of osmotic cues will reduce stored peptide levels in the neurointermediate lobe [15,71]. However, as the dromedaries used in our studies were fully hydrated prior to slaughter, no changes in circulating levels of AVP or OT were observed or expected (Fig 14).

Comparison of our transcriptome data with the literature revealed additional insights into seasonal functionality of the camel supraoptic nucleus. For example, the apelin receptor (APLNR) is up-regulated in summer compared to winter. This receptor mediates the actions of apelin in the supraoptic nucleus, which increases the firing rates of AVP cell, but had no effect on the firing rate of OT neurons [72–74].

Our comprehensive morphological, transcriptomic and peptidomic analysis of the dromedary HNS strongly supports the concept that adaptive changes are taking place that prepare the animal for the prospect of life-threatening challenge in the hot, dry summers of the desert. It is clear that, in addition to changes in neuropeptide synthesis, including AVP, its principal neuroendocrine product, the supraoptic nucleus undergoes dramatic remodelling events that presumably facilitate hormone synthesis and secretion. The molecular details and dynamics remain to be elucidated.

## Methods

### Animals

Healthy, age-matched (6–10 years old as determined from dentition) adult male dromedary camels (*Camelus dromedarius*) of the Tergui breed were slaughtered for human consumption in the winter season (22/01/2014; El Oued, Algeria) and in the summer season (23/06/2014; Ouargla, Algeria). See S1 Fig.

### Tissue harvesting

Immediately after slaughter, the SON was rapidly dissected from the brain and immersion fixed in 10% (v/v) formaldehyde or 2.5% (w/v) glutaraldehyde for light or electron microscopy studies respectively. Both fixatives were buffered in phosphate (0.1 M, pH 7.4) at 4°C. Tissue for RNAseq was snap frozen and stored in RNAlater. For mass spectrometry peptide analysis, neurointermediate lobe and supraoptic nucleus samples were collected on ice between 15–20 min after animal slaughter, and frozen for shipping. See S1 Fig.

### Histology and immunohistochemistry

Sections of 10 μm were used. For histology, sections were stained by cresyl violet (Nissl), haematoxylin and eosin, or toluidine blue. For immunhistochemistry, endogenous peroxidase activity was eliminated by incubating tissues with 0.3% (v/v) H_2_O_2_ in methanol for 10 min at room temperature, then non-specific reactivity was blocked by incubation with 3% (v/v) normal goat serum. Tissues were permeabilised in 0.5% (v/v) Triton X-100 for 1h, then incubated separately for 15 h at 4°C with buffered primary antibodies (rabbit polyclonal anti-vasopressin, Ab1567, Merck; rabbit polyclonal anti-oxytocin, Ab911, Merck; mouse monoclonal anti-vimentin, VIM3B4, DAKO, USA). Tissues were subsequently incubated with biotinylated secondary anti-rabbit antibodies for 2 h at room temperature, then reacted with streptavidin-or anti-mouse peroxidase for 2 h at room temperature. Staining was revealed by incubation with DAB solution for 20 min. Control sections were processed in the same way, but without incubation with the primary antibodies. Negative controls were performed by omitting the primary antibody; no immunoreaction was evident.

### Electron microscopy

For electron microscopy, ultrathin sections cut at 80 nm were postfixed in buffered 2% (w/v) osmium tetroxide solution, then dehydrated in graded ethanol and propylene oxide, and finally embedded in Spurr’s resin. They were then placed on gold grids and double-stained with uranyl acetate and lead citrate. Images were obtained using a transmission electron microscope (JEOL 1010).

### Statistical analysis of images

The ultrastructural seasonal variations in the dromedary supraoptic nucleus were analyzed using SYSTAT12 (Systat 12, Version 12.00.08, Systat Software Inc., Chicago, IL, USA). Three elements were quantified each season: 1. Capillaries (area of lumen, Ca; thickness of basal lamina, BLt; Number of fenestrations, Fn; area of fenestrations, Fa). 2. Synapses (synaptic membrane apposition length, SMAl; post-synaptic density length, PSDl; post-synaptic density-area, PSDa) [75]. 3. Number and kind of debris (number of degenerating bodies, DBn; number of degenerating axons, DAn). For quantitative studies, six animals were considered (3 from winter and 3 from summer), and 10 photomicrographs (area 42.25 μm^2^) per animal) were used to calculate element parameters by Mac Biophotonics Image J and Image Tool-IT300 software. The comparison of ultrastructural element parameters between seasons was performed by General Linear Model (GLM); the significant contribution was retained at 5% threshold probability.

### The sequence of the dromedary Targui camel genome

We sequenced the genome of a healthy adult male dromedary camel of the Targui breed. “Jamal” was slaughtered for human consumption in the El Goléa slaughterhouse, situated in the central Algerian Sahara (The Algeria genome; https://www.ncbi.nlm.nih.gov/bioproject/PRJNA310822). See S1 Fig. DNA from the bladder was extracted using DNeasy blood and tissue kit (Qiagen) and eluted into EB buffer (Qiagen) according to manufacturer’s protocols. Resulting DNA samples were purified by ethanol precipitation and the quality confirmed by means of Qubit fluorometer and Nanodrop spectrophotometer. Three libraries corresponding to different insert sizes were then prepared for whole genome sequencing. Firstly, a library was prepared from 2μg DNA using the TruSeq DNA PCR-Free Sample Preparation Kit (TSPF), using the manufacturer’s protocol for 550bp insert size. The second and third libraries were prepared using Nextera Mate Pair Sample Preparation Kit (using Gel-Plus workflow), with 4.5μg input DNA per library. Using the Pippin Pulse user guide, DNA fragments from 3-5kb and 8-10kb were selected from the agarose gel and extracted using Promega Wizard SV Gel and PCR Clean-Up System. A total of 400ng of DNA was recovered from the 3-5kb region band and 180ng of DNA from the 8-10kb region, and these were run according to the NexteraMatePair (NMP) user instructions. The libraries were distributed across 8 lanes of an Illumina HiSeq flow cell, with Illumina PhiX control library spiked into each lane (1. TSPF. 5% PhiX in Lane 1, 1% PhiX in Lanes 2–4; 2. NMP3, 1% PhiX in Lanes 5–6; 3. NMP8. 1% PhiX in Lanes 7–8).

PhiX genome sequences were discarded, sequencing adapters and low quality bases found in all clean reads were trimmed off, and remaining reads shorter than 25 bases were discarded. Three separate strategies were compared to assemble high quality reads from all three libraries:

1. SOAP (Short Oligonucleotide Analysis Package; [76]; http://soap.genomics.org.cn). We first performed base error correction on the high quality sequencing data using SOAPec version 2.03. The error corrected reads were then assembled and scaffolded using SOAPdenovo version 2.04. The resulting scaffold sequences were refined with GapCloser (SOAPgc) for SOAPdenovo version 1.12.
2. Velvet ([77]; https://www.ebi.ac.uk/~zerbino/velvet/). We used all the good quality reads to perform sequence assembly with Velvet de novo assembler version 1.2.10.
3. CLCGWB (CLC Genomics Workbench; CLC bio, Aarhus, Denmark; http://www.clcbio.com/products/clc-genomics-workbench/). We used all the good quality reads to perform de novo sequence assembly and scaffolding with CLC Genomics Workbench version 7.5.0.

Of the three approaches employed, the SOAP scaffolds best represent the dromedary genome, with fewer and larger scaffolds as assessed by validation tests (QUAST, [78]; http://bioinf.spbau.ru/quast. CEGMA, [79]; http://korflab.ucdavis.edu/datasets/cegma/). We then used CLC Genomics Workbench scaffolds to enhance contiguity and correctness of the SOAP scaffolds using GAM-NGS ([80]; https://github.com/vice87/gam-ngs).

Systematic genome analysis enabled the identification of repeats (Repeat Masker and Repeat Modeller for custom *de novo* library, http://www.repeatmasker.org), the alignment of ESTs and protein coding sequences to the genome (Exonerate, [81]; https://www.ebi.ac.uk/~guy/exonerate/. Blastx, [82]; http://blast.ncbi.nlm.nih.gov/Blast.cgi), training of *ab initio* gene predictions (SNAP, [83]; http://korflab.ucdavis.edu/software.html. Augustus, [84]; http://bioinf.uni-greifswald.de/augustus/. GeneMark, [85]; http://exon.gatech.edu/GeneMark/. Exonerate, [81]; https://www.ebi.ac.uk/~guy/exonerate/), and the synthesis of all of these data into gene annotations with evidence-based quality values (MAKER2, [86]; http://www.yandell-lab.org/software/maker.html). The finalized transcript and protein predictions were subjected to functional analysis and pattern search using Blast and Interproscan against the following databases: UniProt Knowledgebase (http://www.uniprot.org); Gene3D (http://gene3d.biochem.ucl.ac.uk/Gene3D/); Panther (http://pantherdb.org); Pfam (http://pfam.xfam.org); SUPERFAMILY (http://supfam.cs.bris.ac.uk/SUPERFAMILY/). Genome metrics are presented in S5 Table.

### RNAseq analysis of the dromedary supraoptic nucleus

Tissues (n = 2 for each season) were extracted in a Category II fume hood. Samples were weighed and dissected to ensure that each was less than the 30mg to ensure that extraction by column was maximally efficient. Each sample was then homogenized in 1ml Trizol and the aqueous phase separated with Chloroform. Purification relied on the RNeasyMiniKit (Qiagen) according to manufacturers protocol. Concentration and quality of RNA was determined using both Nano-drop and Agilent Bioanalyzer 2100 and samples with a R.I.N>8 were used for library construction. Stranded total RNA libraries were prepared according to IlluminaTruSeq stranded total RNA sample preparation guide (April 2013, Rev D) for Illumina Paired-End Multiplex Sequencing (Source Bioscience, Nottingham, UK). Ribo-Zero gold kit was used to remove cytoplasmic and mitochondrial rRNA prior to fragmentation and priming for first and second strand cDNA synthesis. The 3’ ends of fragments were adenylated and adapters and indexes (for the multiplex barcoding) ligated. Libraries were then subject to further quality control prior to pools with a concentration of 8pM being loaded onto lanes of an Illumina flowcell and sequenced using 100bp Paired End runs. Each sample resulted in >35million reads.

### RNAseq data analysis

Our analysis consisted of RNA-Seq alignments for every sample followed by differential expression (DE) prediction that generates tables of adjusted p-values for identifying genes with significant DE. We used Tophat to map RNA-Seq reads to our dromedary genome assembly [87]. Tophat’s default settings are optimised for the human genome, so we first adjusted these settings according to the genome of interest. We adjusted this range of acceptable intron lengths by using gene models to estimate the distribution of intron lengths and by selecting a range of sizes that account for 99.9% of known introns. In the dromedary genome, more than 3,000 putative introns have lengths that fall below while none are longer than 100,000; our final range was set to 8–52,000. A key aspect of our analysis was predicting genes that are up- or down-regulated under the different conditions (winter vs. summer). We used HTSeq [88] to generate read counts from each BAM file and used bespoke scripts to merge the counts into tables for DE prediction using DESeq [89] and edgeR [90]. Significance testing is pairwise via Fisher's Exact Test which is optimal for experiments with low replicates. Genetic heterogeneity of our samples precluded multiple testing correction and we instead relied upon peptidome analysis to establish biological plausibility. Comparisons with average read-counts of less than 10 in both the summer and winter samples were excluded. Gene ontology tool PANTHER (Protein Analysis THrough Evolutionary Relationships, http://pantherdb.org; [22]) was used examine significant genes. Given the input to this analysis was based on genes with an unknown false discovery rate, we only pursued functions with biological plausibility. Raw data has been banked (GSE131361 study at:

https://www.ncbi.nlm.nih.gov/geo/query/acc.cgi?acc=GSE131361).

### Extraction of peptides

Frozen supraoptic nucleus tissue was placed individually in 1001 μL of 15 mg/ml of 2,5-dihydroxybenzoic acid (DHB) aqueous solution, while neurointermediate lobe were placed in 500 μL each and shipped to the University of Illinois UI. At UI, neurointermediate lobe samples were diluted to 1.5 mL using identical DHB solution and incubated for 48h as one-step extraction procedure described elsewhere [91]. The tissue extract samples were grouped as follows: group S–summer (n = 3), W–winter (n = 4). Neurointermediate lobe samples ranged in size 44–77 mg for summer samples, and 65-152mg for winter samples. Supraoptic nucleus samples were more uniform.

### Measurement of the neurointermediate lobe and supraoptic nucleus peptide profiles by matrix-assisted laser desorption/ionization time-of-flight mass spectrometry

For measurement of peptide profiles, 0.7 μL of the supraoptic nucleus DHB extraction solution was spotted on a stainless-steel MALDI TOF MS target and co-crystallized with 0.7 L of freshly prepared concentrated DHB matrix (50 mg/mL 50% v/v acetone). Neurointermediate lobe DHB extracts were first filtered through Amicon filters with MWCO 10K to eliminate large proteins, and processed for MALDI TOF MS sampling as described above for supraoptic nucleus samples. Positive ion mass spectra from duplicate technical replicates of each neurointermediate lobe and supraoptic nucleus extract samples were acquired manually at 1 KHz laser frequency and laser constant power optimized for the sample type in the m/z 600–6000 region using a Bruker ultrafleXtreme mass spectrometer (Bruker Daltonics, Bremen, Germany) operated in reflectron mode. Acquisition parameters included positive ion mode, 500 laser shots per raster step, multiple steps over each sample spot, accumulation of 10,000 laser shots per sample. External quadratic calibration was adjusted for every 5x5 sample spot square using Bruker Peptide Mix II (Bruker Daltonics, Bremen, Germany).

### Principal component analysis of supraoptic nucleus and neurointermediate lobe peptide profiles

Statistical analysis of raw MALDI TOF MS data was performed using ClinProTools 3.0 software (Bruker Daltonics, Bremen, Germany). All spectra were normalized to total ion count (TIC), Level scaled, and processed for TopHat baseline correction (1%) within 800–5000 m/z, smoothed to average isotopic clusters using 4 cycles of Savitzky–Golay filter over 2 m/z range and grouped by animal ID and then by season. Winter group consisted of 8 spectra from 4 winter samples, Summer group consisted of 6 spectra from 3 summer samples. Automatic peak selection was always performed on the total average group spectrum for each season. Peaks with signal to noise ratio greater than 5 and above 1% relative intensity threshold were selected on average group spectrum for each season. Manual inspection/editing of automatic peak integration was done on the mean spectrum representative of each season group in order to ensure that entire isotopic clusters of highly resolved peaks were included. Peptide profiles of mean spectra were compared by principal component analysis followed by Anderson-Darling (AD) normality test and *t*-test for normal distributed data. Data not showing normal distribution (pAD ≤0.05) were evaluated by Wilcoxon or Kruskal-Wallis test, respectively. To decrease the number of false positives while computing individual peak statistics on the complex spectra, the Benjamini-Hochberg procedure incorporated into ClinProTool was automatically applied for p-value adjustment during analysis [91]. Unsupervised clustering of spectra was performed on principal component analysis-modified data using Euclidean distance, Average distance methods and Minkowski exponent of 1.5. Performance of a binary classification was assessed by plotting receiver operating characteristic curves for all picked peaks.

### Peptidomic characterization of supraoptic nucleus

For first stage purification by high performance liquid chromatography (Breeze II, Waters, Milford, MA, USA), 25 μL-portions of each summer or winter supraoptic nucleus extract were combined by season into 75–100 μL sample and loaded separately onto Grace Vydac C18 column, 5μm particle size, 2.1 x 15 mm, at uniform flow rate of 250 μL/min and starting conditions of 2% solvent B. Solvent A was 5% ACN/0.1% (v/v) HFBA (heptafluorobutyric acid), solvent B 95% ACN/0.1% (v/v) HFBA. Entire separation was 70 min, analytical gradient 2–45% (v/v) B was developed over 45 min with brief isocratic step at 20%B for removal of bulk of DHB. Fractions were collected every 2 min, dried in vacuum concentrator, reconstituted in 10 μL of 0.1% FA and assessed for peptide presence by MALDI TOF MS. Fractions containing peptides were subjected to structural characterization by liquid chromatography-tandem mass spectrometry using Thermo Ultimate 3000 RSLC (Thermo Scientific, Sunnyvale, CA, USA) with pre-column trap and nanoflow selector hyphenated to Bruker Impact HD mass spectrometer via Bruker CaptiveSpray source (Bruker Daltonics, Bremen, Germany). Sample volume was optimized for each fraction and loaded onto Acclaim PepMap μ-precolumn at 15 μL/min of loading solvent (0.1% FA/0.01% TFA). After 3 min, precolumn was put in-line with Acclaim PepMap100 C18 analytical column (3 μm, 100 Å, 75 μm i.d. x 15 cm). Peptides were fractionated using 0.1% FA as solvent A and 80% ACN/0.1% FA as solvent B at 300 nL/min over 80 min concave gradient from 2% to 50% B; the entire run was 120 min. The MS data were collected for a full scan at 1 Hz, MS/MS scan rate was automatically adjusted at 1–4 Hz rate depending on signal intensity. Dynamic precursor ion exclusion was applied to top 8 ions for 1 min after 2 spectra.

### Bioinformatic identification of prohormones and peptides in supraoptic nucleus

Raw tandem mass spectrometry spectra were loaded into DataAnalysis software v4.2 Bruker Daltonics, Bremen, Germany), processed for base peak chromatogram extraction, compound spectra calculation, charge deconvolution, and exported as mascot generic files (mgf). The mgf files were loaded into PEAKS Studio 8.0 (Bioinformatics Solutions Inc, Waterloo, ON, Canada) and processed for de novo sequencing and database search algorithms using the following parameters: mass tolerance—20 ppm precursor ion, 0.5 Da fragment ion, no enzyme; post-translational modifications—acetylation (K), acetylation (N-terminus), amidation, oxidation (M), half-disulfide bridge (C), pyroglutamylation (Q, E). The NCBI dromedary translated protein database (ftp://ftp.ncbi.nlm.nih.gov/genomes/Camelus_dromedarius/protein/, 26728 sequences) as well as custom-annotated protein database based on our RNAseq data (69328 sequences) were used. Automatic peptide hits were filtered at 1% false discovery for peptide-spectrum match, after which matched peptide sequences and protein assignments were manually curated. The mass spectrometry proteomics data have been deposited to the ProteomeXchange Consortium (http://www.proteomexchange.org) via the PRIDE partner repository (https://www.ebi.ac.uk/pride/archive/) with the dataset identifier PXD013869 and 10.6019/PXD013869. SignalP 4.1 (http://www.cbs.dtu.dk/services/SignalP/) [92] was used for prediction of signal peptides, and Neuroped (http://neuroproteomics.scs.illinois.edu/neuropred.htm) [93] aided interpretation for cross-referencing peptide assignment between liquid chromatography-mass spectrometry/mass spectrometry and MALDI TOF MS data.

### Plasma AVP/OT measurement by radioimmunoassay

The extraction of AVP and OT hormones was performed using 1 ml of dromedary plasma with acetone and petroleum ether and the hormones were measured by specific radioimmunoassay techniques as described [29,94,95]. Assay sensitivity and intra- and inter-assay coefficient of variation were 0.1 pg/mL, 2.9% and 4.8% for AVP, 0.1 pg/mL, 3.5% and 11.5% for OT.

## Supporting information

S1 Fig Experimental strategy. (A and B) The brain of the dromedary camel in ventral view demonstrating the anatomical position of the HNS. (A) Pituitary (Pt) attachment to brain before removing. Inset–diagramatic representation illustrating the relative positions of the hypothalamus and the Pt, which consists of the anterior pituitary and the neurointermediate lobe of the pituitary (NIL). (B) Third ventricle hole (light asterisk) after removal of the pituitary and its neurointermediate lobe (dark asterisk) (inset). The part of the brain containing the SON is indicated (dashed line rectangle). The supraoptic nucleus was subjected to electron microscopic, transcriptomic and peptidomic analysis. The neurointermediate lobe was the subject of peptidomic analysis. (C) Diagramatic representation of the hypothalamo-neurohypophyseal system (HNS) showing axons originating in the supraoptic nucleus (SON) of the hypothalamus projecting to the posterior (neural) lobe of the pituitary. (D) Structure of the HNS. Within the cell body of the magnocellular hypothalamic neuron, AVP mRNA is translated into a prepropeptide which enters the ER. After signal peptide cleavage, the propeptide is sorted into the regulated secretorypathway. Passage through the Golgi and trans-Golgi network is accompanied by packaging into dense core granules and processing into mature bioactive peptides. The granule is transported down the axon to storage in axon terminals located in the posterior pituitary. Release into the circulation is elicited by neuronal inputs governed by physiological stimuli. Within the posterior pituitary there is an intimate and physiologically important relationship between the axon terminal, the blood vessel and specialised glial cells called pituicytes. (E) Sites in the Algerian Sahara desert where camel tissues were harvested. (F) We sequenced the genome of “Jamal”. (G) We compared camels in winter and summer by electron microscopy (winter, 3; summer 3); RNAseq (winter, 2; summer 2) and mass spectrometry (winter, 4; summer 3).
(TIF)

S2 Fig Coronal hypothalamic sections at the level of the optic chiasm and its tract. Slices obtained rostro-caudally (A, B, C and D) and their corresponding Nissl stained histological sections (A', B', C' and D’). Asterisks indicate the supraoptic nucleus (SON) zones. A magnified zone from the SON shows magnocellular neurone (MN) cell bodies (inset in C). OC: optic chiasm; op.t: optic tract; 3V: third ventricle; ME: Median eminence; P.tb: pars tuberalis; Scale bars A, B, C, D = 5 mm; A', B', C', D' = 200 μm.
(TIFF)

S3 Fig General organization of the supraoptic nucleus (SON). (A) Micrographs demonstrating the most striking elements at the nucleus, vessels (arrows) and magnocellular neurone perikaryons (blue dots). (B) Magnocellular neurons (arrowheads), capillaries (dark arrows) and glial cells (light arrows) are the most important elements observed in SON at this level of magnification (haematoxylin and eosin stain). (C, D) Micrographs from semi-thin sections, presenting the somatic (Sz) and dendritic zones (Dz) of the SON. (C) The Sz contains magnocellular neurone perikaryons, appearing as dark (dark arrowheads) and light (light arrowheads) profiles (C and D). Glial cells (light arrows), capillaries (dark arrows) and MNs are distribution across the nucleus (D) (Toluidine blue stain). Scale bars A = 100 μm; B = 10 μm; C = 20 μm; D = 10 μm.
(TIFF)

S4 Fig Immunohistochemical staining of neuronal and glial components of the SON. (A) Vasopressinergic (AVP) magnocellular neurones. (B) Oxytocinergic (OT) magnocellular neurones (inset–higher magnification of dashed square). (C) Glial processes immunolabeled by anti-vimentin (arrows). Asterisks indicate magnocellular neurones. op.t: optic tract nerve. Scale bars A = 100 μm; B = 100 μm; C = 10 μm. Negative controls were performed by omitting the primary antibody; no immunoreaction was evident.
(TIFF)

S5 Fig Identification of select peptides from the camel NIL extracts by tandem mass spectrometry. Annotated spectra show detected ion series (*b*-blue, *y*-red) and fragmentation of the peptide sequence. (A) vasopressin; (B) des-acetylated melanotropin alpha, a-MSH; (C) melanotropin alpha, a-MSH.
(TIF)

S6 Fig AVP in the camel NIL and SON. A) Representative MALDI-TOF MS spectra from camel NIL and SON showing AVP detection (arrow). (B) Confirmation of AVP assignment in both cyclic (m/z 1084.445) and linear (m/z 1086.445) forms by comparing theoretical isotopic patterns of matching masses in NIL (top trace) and SON (bottom trace). Dotted traces represent theoretical isotopic pattern for cyclic peptide (grey) and linear peptide (blue).
(TIF)

S7 Fig Seasonal differences in detection of TAC1-derived peptides by LC-MS/MS. Purple highlights peptides detected only in winter, blue indicates peptides detected only in summer.
(TIF)

S1 Table Global catalogue of gene expression in the dromedary camel supraoptic nucleus comparing summer and winter. Average reads for summer and winter were determined using DESeq. Fold change between summer and winter and P values were calculated using edgeR.
(CSV)

S2 Table Robust analysis of our transcriptome data revealed 171 differentially regulated genes (>2 fold difference; p<0.05, n = 2 for each season). Of these, 112 gene transcripts are present at a higher level in winter compared to summer, whilst 59 mRNAs are more prevalent in summer compared to winter.
(CSV)

S3 Table Supraoptic nucleus peptide identification using liquid chromatography-tandem mass spectrometry and identified via automatic de novo spectra interpretation followed by de novo tag search using the *Camelus dormedarius* protein database from NCBI ftp://ftp.ncbi.nlm.nih.gov/genomes/Camelus_dromedarius/protein/. (XLSX)

S4 Table Seasonal differences in peptides identified in the dromedary camel supraoptic nucleus by tandem mass spectrometry. (XLSX)

S5 Table Dromedary camel genome assembly metrics. (DOCX)
