# Supplementary figures and images for: Seasonal adaptations of the hypothalamo-neurohypophyseal system of the dromedary camel
Source: PLoS One. 2019 Jun 18;14(6):e0216679. doi: 10.1371/journal.pone.0216679 (PMC6581255; doi:10.1371/journal.pone.0216679)

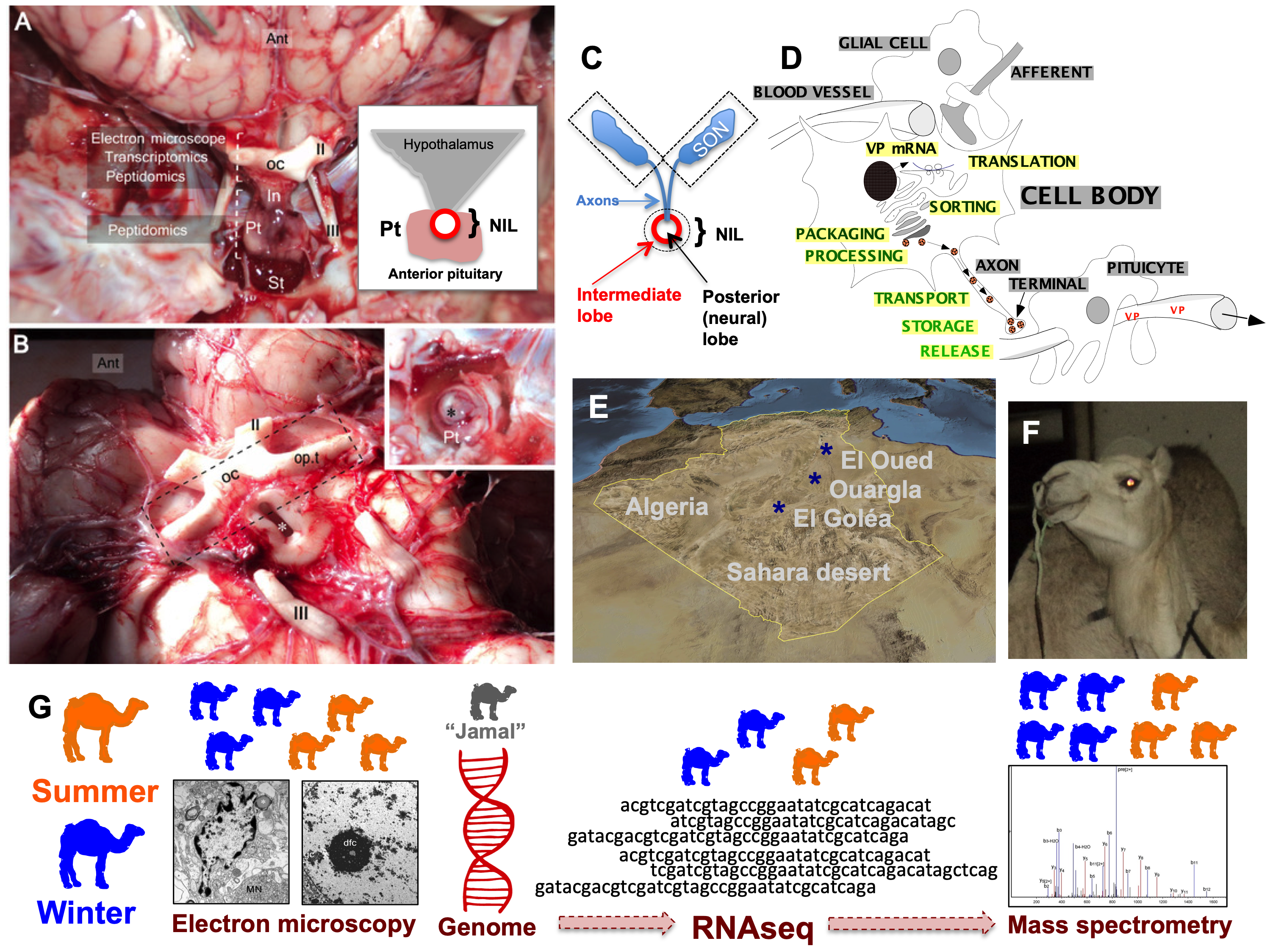

Supplement: S1 Fig — (A and B) The brain of the dromedary camel in ventral view demonstrating the anatomical position of the HNS. (A) Pituitary (Pt) attachment to brain before removing. Inset–diagramatic representation illustrating the relative positions of the hypothalamus and the Pt, which consists of the anterior pituitary and the neurointermediate lobe of the pituitary (NIL). (B) Third ventricle hole (light asterisk) after removal of the pituitary and its neurointermediate lobe (dark asterisk) (inset). The part of the brain containing the SON is indicated (dashed line rectangle). The supraoptic nucleus was subjected to electron microscopic, transcriptomic and peptidomic analysis. The neurointermediate lobe was the subject of peptidomic analysis. (C) Diagramatic representation of the hypothalamo-neurohypophyseal system (HNS) showing axons originating in the supraoptic nucleus (SON) of the hypothalamus projecting to the posterior (neural) lobe of the pituitary. (D) Structure of the HNS. Within the cell body of the magnocellular hypothalamic neuron, AVP mRNA is translated into a prepropeptide which enters the ER. After signal peptide cleavage, the propeptide is sorted into the regulated secretorypathway. Passage through the Golgi and trans-Golgi network is accompanied by packaging into dense core granules and processing into mature bioactive peptides. The granule is transported down the axon to storage in axon terminals located in the posterior pituitary. Release into the circulation is elicited by neuronal inputs governed by physiological stimuli. Within the posterior pituitary there is an intimate and physiologically important relationship between the axon terminal, the blood vessel and specialised glial cells called pituicytes. (E) Sites in the Algerian Sahara desert where camel tissues were harvested. (F) We sequenced the genome of “Jamal”. (G) We compared camels in winter and summer by electron microscopy (winter, 3; summer 3); RNAseq (winter, 2; summer 2) and mass spectrometry (w [file pone.0216679.s001.tif]

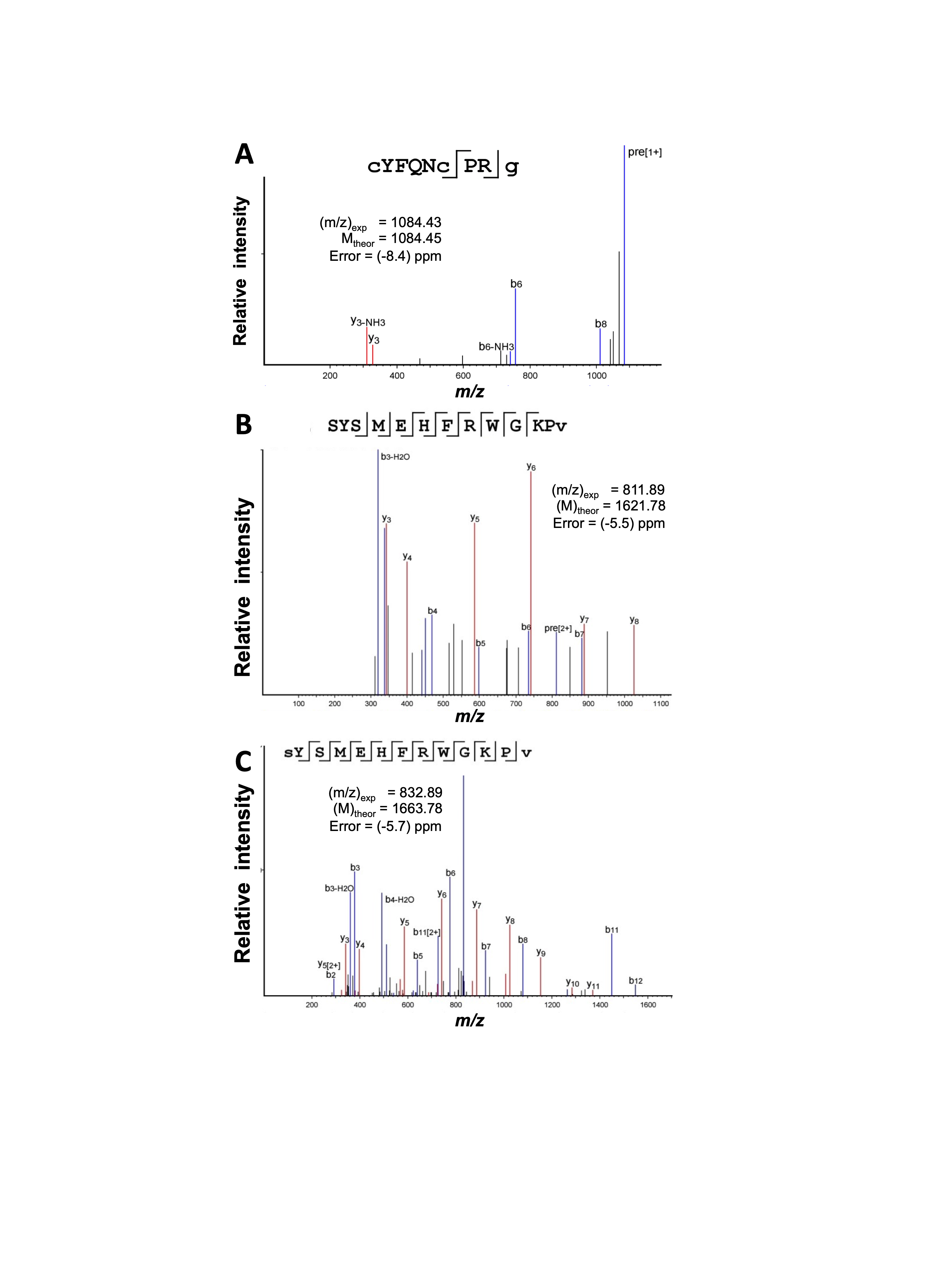

Supplement: S5 Fig — Annotated spectra show detected ion series (b-blue, y-red) and fragmentation of the peptide sequence. (A) vasopressin; (B) des-acetylated melanotropin alpha, a-MSH; (C) melanotropin alpha, a-MSH. (TIF) [file pone.0216679.s005.tif]

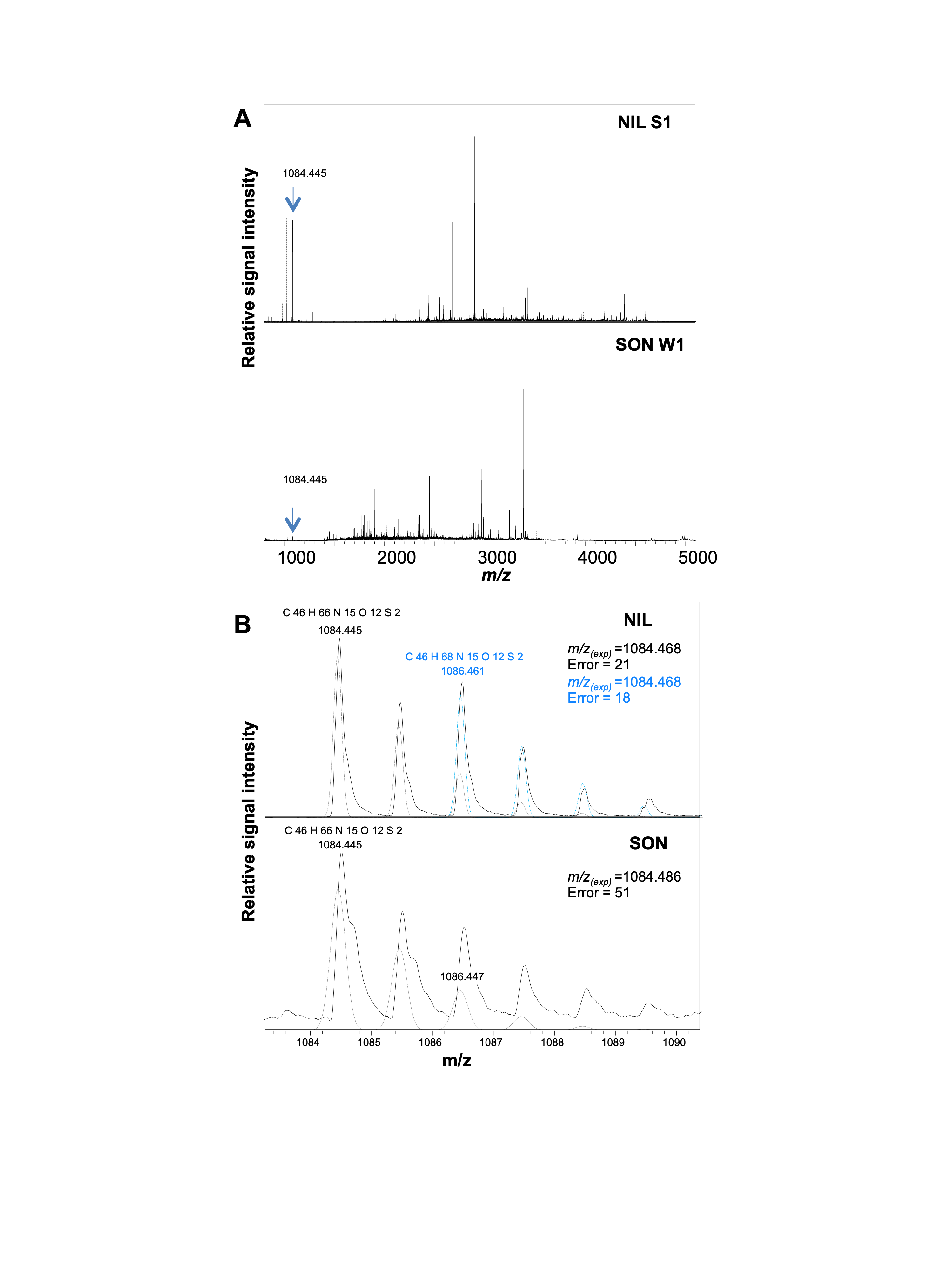

Supplement: S6 Fig — A) Representative MALDI-TOF MS spectra from camel NIL and SON showing AVP detection (arrow). (B) Confirmation of AVP assignment in both cyclic (m/z 1084.445) and linear (m/z 1086.445) forms by comparing theoretical isotopic patterns of matching masses in NIL (top trace) and SON (bottom trace). Dotted traces represent theoretical isotopic pattern for cyclic peptide (grey) and linear peptide (blue). (TIF) [file pone.0216679.s006.tif]

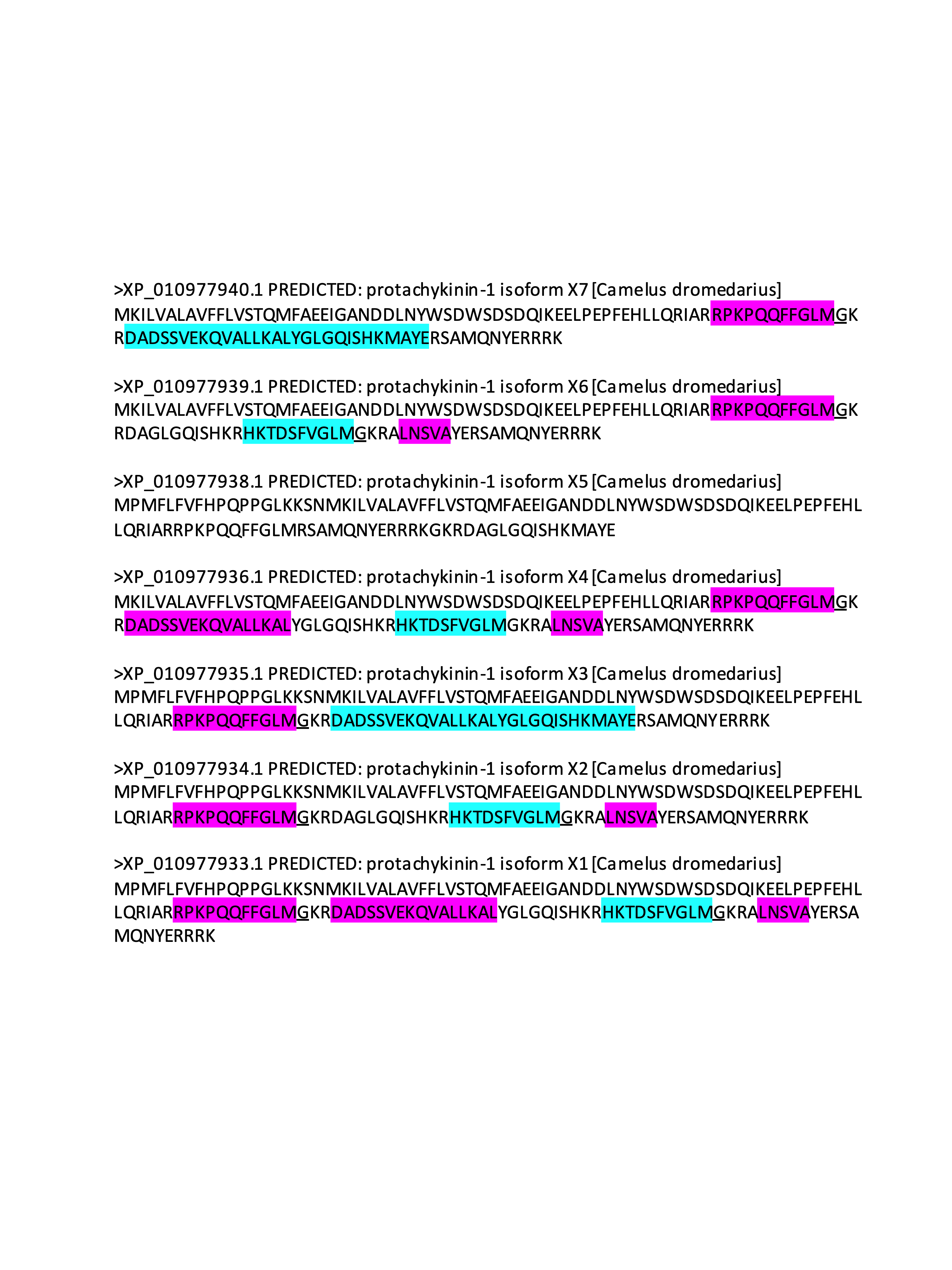

Supplement: S7 Fig — Purple highlights peptides detected only in winter, blue indicates peptides detected only in summer. (TIF) [file pone.0216679.s007.tif]
