# Supplementary material for: Seasonal adaptations of the hypothalamo-neurohypophyseal system of the dromedary camel
Source: PLoS One. 2019 Jun 18;14(6):e0216679. doi: 10.1371/journal.pone.0216679 (PMC6581255; doi:10.1371/journal.pone.0216679)
Supplement: S5 Table — (DOCX) [file pone.0216679.s012.docx]

**S5 Table. Genome assembly metrics**

| **Genome Assembly Metrics** | |
| --- | --- |
| Number of contigs | 207,871 |
| Assembly length (bp) | 2,084,536,768 |
| Average contig length (bp) | 10,028 |
| Shortest contig length (bp) | 200 |
| Longest contig length (bp) | 282,053 |
| N50 | 31,503 |
| GC% | 41.47% |
| Genome completeness* | 97.58% |
| **Genome Annotation Metrics** | |
| Predicted genes** | 63796 |
| Transcripts | 68006 |
| Exons | 273312 |

*Genome completeness is estimated as the percentage of 248 ultra-conserved CEGs detected in the genome sequence (partial or full length).

**The number of predicted genes in high for two reasons:

1. gene models were predicted on the basis low stringency criteria in order to reveal as many genes/transcripts as possible for RNAseq analysis.

2. In a draft genome assembly such as this, fragmentation or duplication will increase the number of predicted genes.
